# Influence of Incorporation of Different d*^n^*-Electron Metal Cations into Biologically Active System on Its Biological and Physicochemical Properties

**DOI:** 10.3390/ijms222312909

**Published:** 2021-11-29

**Authors:** Bartłomiej Rogalewicz, Małgorzata Szczesio, Ewa Poleszak, Joanna Kowalczyk, Bernadeta Szewczyk, Bruno Cury Camargo, Jacek Szczytko, Marcin Witkowski, Andrzej Fruziński, Anita Raducka, Robert Banasiak, Agnieszka Czylkowska

**Affiliations:** 1 Institute of General and Ecological Chemistry, Faculty of Chemistry, Lodz University of Technology, Zeromskiego 116, 90-924 Lodz, Poland; bartlomiej.rogalewicz@dokt.p.lodz.pl (B.R.); malgorzata.szczesio@p.lodz.pl (M.S.); andrzej.fruzinski@p.lodz.pl (A.F.); anita.raducka@edu.p.lodz.pl (A.R.); 2 Laboratory of Preclinical Testing, Chair and Department of Applied and Social Pharmacy, Medical University of Lublin, Chodzki 1, 20-093 Lublin, Poland; ewa.poleszak@umlub.pl (E.P.); joanna.kowalczyk@umlub.pl (J.K.); 3 Department of Neurobiology, Maj Institute of Pharmacology Polish Academy of Sciences, 12 Smętna Street, 31-343 Kraków, Poland; szewczyk@if-pan.krakow.pl; 4 Institute of Experimental Physics, Faculty of Physics, University of Warsaw, Pasteura 5, 02-093 Warszawa, Poland; Bruno.Camargo@fuw.edu.pl (B.C.C.); Jacek.Szczytko@fuw.edu.pl (J.S.); 5 Faculty of Chemistry, University of Warsaw, Pasteura 1, 02-093 Warszawa, Poland; m.witkowski2@uw.edu.pl; 6 Institute of Applied Computer Science, Faculty of Electrical, Electronic, Computer and Control Engineering, Lodz University of Technology, Stefanowskiego 18/22, 90-001 Lodz, Poland; robert.banasiak@p.lodz.pl

**Keywords:** crystal structure, FTIR spectroscopy, thermogravimetric analysis, imipramine, magnetic studies, central nervous system, Zebrafish

## Abstract

Three new compounds, namely [HL]_2_^+^[CuCl_4_]^2−^, [HL]_2_^+^[ZnCl_4_]^2−^, and [HL]_2_^+^[CdCl_4_]^2−^ (*where L: imipramine*) were synthesized and their physicochemical and biological properties were thoroughly investigated. All three compounds form isostructural, crystalline systems, which have been studied using Single-Crystal X-ray diffraction analysis (SC-XRD) and Fourier-transform infrared spectroscopy (FTIR). The thermal stability was investigated using thermogravimetric analysis (TGA) and melting points for all compounds have been determined. Magnetic measurements were performed in order to study the magnetic properties of the compounds. The above mentioned techniques allowed us to comprehensively examine the physicochemical properties of the newly obtained compounds. The biological activity was investigated using the number of Zebrafish tests, as it is one of the most common models for studying the impact of newly synthesized compounds on the central nervous system (CNS), since this model is very similar to the human CNS.

## 1. Introduction

Imipramine, one of the first successful antidepressant drugs for treating major depressive disorder, was discovered in 1958 [1]. Imipramine is a prototype of the tricyclic class of antidepressants [2]. Following oral administration, it is rapidly and completely absorbed (>95%), with peak plasma concentrations occurring within 2–6 h and readily crosses the blood–brain barrier. Generally, accumulation of imipramine in the brain is about 30–40 times the concentration in plasma. The absorption of imipramine takes place in the small intestine, with little or no absorption occurring in the stomach. Absorption, peak drug concentration, and time to peak do not depend on food consumption. Imipramine is subjected to extensive first-pass metabolism in the liver to yield the primary active metabolite (desipramine). Thus, the therapeutic effect of imipramine depends also on the amount of desipramine formed [1].

For a long time, imipramine was the drug of choice for treating depression, especially in the more severe forms of the disease. Unfortunately, the drug has several adverse effects, including dry mouth, constipation, urinary retention, blurred vision, palpitations, and tachycardia [2]. Despite continuous works on creating new antidepressants, side effects remain a considerable problem. Therefore, several supplementations have been employed with different antidepressants to reduce drug dose and decrease adverse effects while maintaining therapeutic effects. Such preclinical and clinical studies have also been conducted with imipramine. Recent data revealed that zinc enhances the efficacy/potency of imipramine in preclinical paradigms sensitive to antidepressants. Sub-effective doses of zinc combined with sub-effective doses of imipramine resulted in antidepressant-like effects in the forced swim test, tail suspension test, and chronic unpredictable stress model of depression [3,4,5]. Moreover, clinical studies indicate that zinc supplementation augments the efficacy and speed of onset of the therapeutic response to imipramine, especially in patients non-responsive to antidepressant pharmacotherapies [6].

The compounds that we applied in the present project are meant to influence the central nervous system (CNS) by decreasing depression and anxiety symptoms. They are derivatives of imipramine, the well-known tricyclic antidepressant that reduces the serotonin and noradrenaline reuptake, and metallic cations (Cu^2+^, Zn^2+^, and Cd^2+^) that were supposed to enhance the imipramine activity. Since the compounds were newly synthesized, the screening of the activity towards the CNS and the toxicity test were performed using the Zebrafish model. In the present study, we have used diazepam as a reference standard for the evaluation of an anxiolytic activity. There are also studies on Zebrafish larvae proving that diazepam increases the level of serotonin and tryptophan in a dose-dependent manner after 2.5 h of bathing in the tested solution [7].

Zebrafish (*Danio rerio*) model has gained popularity in neuropharmacological research due to its high physiological and genetic homology to humans, as well as similar CNS morphology. Zebrafish genes show 70% correspondence to humans’ nucleotide sequence and a similar number of chromosome pairs equal to 25, whereas humans have 23. That gives the advantage for Zebrafish compared to rats and mice which have 21 and 20 pairs of chromosomes, respectively. Moreover, Zebrafish possess genes that are counterparts of those in humans responsible for human diseases [8,9]. For the presented project, the Zebrafish model was used since their rapid development allows evaluating the toxicity profile and performing a fast screening of the tested compounds. The transparency of embryos in the early days and their rapid growth enable following developmental disruptions and assessing whether a newly synthesized drug is safe for the living creatures or on which stage of development it is toxic [10]. Moreover, there is high cost-efficiency as well as established anxiety-like and locomotor activity behavioral tests [11].

The amygdala and habenula, which regulate the release of serotonin and dopamine, are responsible for affective behaviors in both Zebrafish and humans. The hyperactivity of habenula was detected in patients with depression as well as in rodents and Zebrafish with anxiety/stress-like behaviors. Interestingly, reaction to stress in Zebrafish and mammals is similar and engages the cortisol mediation on the axis of the hypothalamus–pituitary gland acting on glucocorticosteroid receptors [12,13,14]. Studies show that mood disorders in the Zebrafish model were associated with reduced activity and chronic elevation of cortisol and reversed by reserpine and d-amphetamine [13,15]. We applied the locomotor protocol with the phases of light and dark in order to create stressful conditions for Zebrafish five-day larvae [16]. The measurement of locomotor activity in light/dark transition may be a useful tool for preclinical drug screening of potential anxiolytic/antidepressive drugs. The study by Giacomini showed that fluoxetine, the selective serotonin reuptake inhibitor, and tryptophan, the amino acid precursor of serotonin, decreased anxiogenic effects in adult Zebrafish by decreasing distance traveled, entries to the top of the tank, or by increasing time spent in the top of the tank and the cortisol levels compared with the control group [17]. The association of serotonin system dysfunctions and depression with a high level of cortisol and stress is well-established. Moreover, in Zebrafish, all major neuromodulatory systems similar to those in humans and rodents were found. They contain neurotransmitter receptors, transporters, and enzymes of synthesis and metabolism such as tyrosine hydroxylase essential for catecholamines synthesis [18]. It has been proved that Zebrafish have serotonin receptors such as 5-hydroxytryptamine receptor 1A (htr1aa), the expression of which can be modulated by different antidepressant agents or even therapy with supplementation of guts microbiota [19].

Taken together, it was reasonable to perform the presented study on the Zebrafish model to obtain strong grounds for the following investigations of the synthesized compounds.

## 2. Results and Discussion

### 2.1. Synthesis

[HL]_2_^+^[CuCl_4_]^2−^ compound: 136 mg of copper(II) chloride dihydrate (0.8 mmol) was dissolved in 5 mL of ethanol; 507 mg of imipramine hydrochloride (1.6 mmol) was dissolved in 20 mL of ethanol and slowly added to the solution of copper(II) chloride. The reaction was carried out at room temperature with constant mixing with a magnetic stirrer. After a few moments, a gold-colored, crystalline precipitate was formed. After 2 h, the precipitate was filtered and washed several times with small amounts of ethanol. Then, the product was dried in the open air and analyzed.

[HL]_2_^+^[ZnCl_4_]^2−^ compound: 109 mg of anhydrous zinc(II) chloride (0.8 mmol) was dissolved in 5 mL of ethanol; 507 mg of imipramine hydrochloride (1.6 mmol) was dissolved in 20 mL of ethanol and slowly added to the solution of salt. The reaction conditions were the same as for the previous compound. After a few moments, a white, crystalline precipitate was formed. As previously, after 2 h, the precipitate was filtered and washed several times with small amounts of ethanol. The resulting compound was analyzed after drying in the open air.

[HL]_2_^+^[CdCl_4_]^2−^ compound: 55 mg of anhydrous cadmium(II) chloride (0.3 mmol) was dissolved in 40 mL of ethanol; 190 mg of imipramine hydrochloride (0.6 mmol) was dissolved in 10 mL of ethanol and slowly added to the solution of cadmium(II) chloride. After 2 h of mixing at room temperature, a clear solution was left for slow solvent evaporation. As a result, colorless crystals of the product were obtained and later analyzed.

### 2.2. X-ray Diffraction Analysis

**Figure 1:**
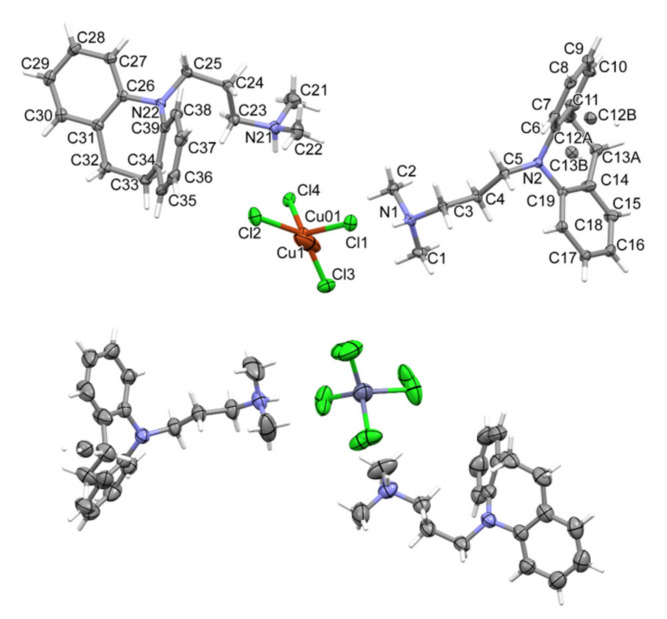
The molecular structures of the [HL]_2_^+^[CuCl_4_]^2−^ compound showing the atom-labelling schemes (above) and the [HL]_2_^+^[ZnCl_4_]^2−^ compound (below).

Tested compounds crystallized in the orthorhombic crystal system in the *Pbca* space group. Crystal data and structure refinement details are summarized in Table 1. The molecular unit, [HL]_2_[MCl_4_] (where M = Cu^2+^ or Zn^2+^) consists of two N,N-dimethyl-3-(1,4,5,6-tetrahydrobenzo[b][1]benzazepin-11-yl)propan-1-amine cations and a tetrahedral [MCl_4_]− anion (Figure 1).

**Figure 2:**
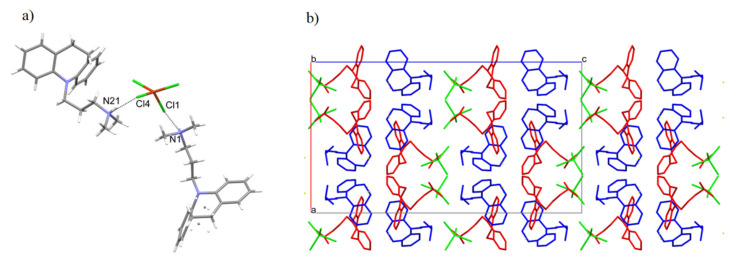
(**a**) The intermolecular hydrogen bonds. (**b**) Packing of the molecules in the crystal of [HL]_2_^+^[CuCl_4_]^2−^ compound along the b axis.

Unit cell parameters indicate that the structures are isostructural (the parameter indicating the similarity of cells is Π = 0.0186 [20]). The copper-containing salt exhibits a disorder at one imipramine molecule and at the copper position. However, in the case of a zinc compound, which was measured at 294 K, there is an identical disorder on the imipramine molecule and additionally on the entire anion (ZnCl_4_^2−^). Both structures have analogous N-H... Cl hydrogen bonds stabilizing the cation–MCl_4_–cation fragment (Tables S1 and S2 and Figure 2).

The symbol for the hydrogen graph-set, according to Berstein’s theory [21], is represented as DD. The packing of molecules in space is characterized by layers of the anion–cation–cation–anion type (Figure 2b). In addition, the compound with cadmium was measured at 99.9 K. The unit cell parameters are 34.8583 Å, 19.2226 Å, 11.4967 Å, 90°, 90°, 90°, and the same space group (Pbca), which may indicate isostructurality.

**Figure 3:**
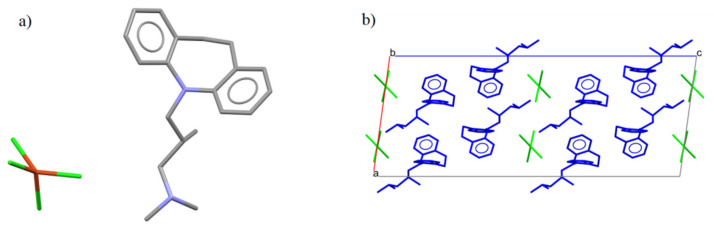
(**a**) The molecular structures of the AFOLOW. (**b**) Packing of the molecules in the AFOLOW along the b axis.

There is an analogous compound in the CSD crystallographic base [22] that differs by an additional methyl group (Figure 3a). The compound AFOLOW [23] adopts an analogous hydrogen bond and packing (Figure 3b).

### 2.3. FTIR Spectra Analysis

**Figure 4:**
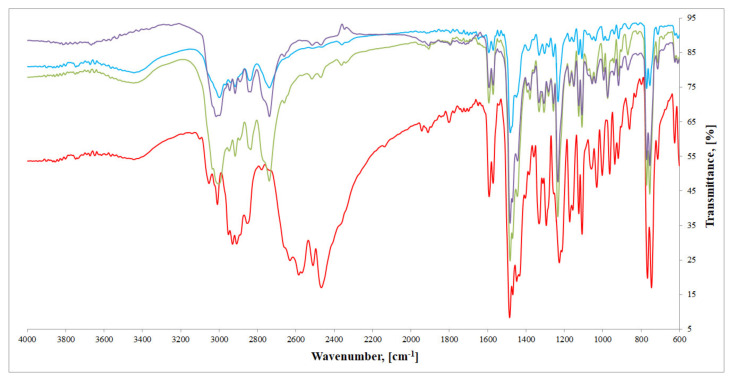
FTIR spectra of investigated compounds: [HL]_2_^+^[CuCl_4_]^2−^ (blue), [HL]_2_^+^[ZnCl_4_]^2−^ (green), [HL]_2_^+^[CdCl_4_]^2−^ (purple), and imipramine hydrochloride (red).

FTIR spectra of all three compounds (Figure 4) are in good agreement with crystallographic data derived from SC-XRD analysis. FTIR spectrum of imipramine hydrochloride (red line) exhibits several important bands in the 3050–2450 cm^−1^ region. These bands can be ascribed to typical *ν*(NH)_amine salt_, as well as *ν*(CH)_aliphatic_ modes: 3049, 3007, 2937, 2927, 2905, 2845, 2769, 2620, 2569, 2507, 2460 cm^−1^. In all three compounds’ spectra, described bands are either not present, or significantly moved, as a consequence of imipramine–metal cation interactions described in the crystallographic section, mainly hydrogen bonds. These bands, however, are altered in the same manner, which confirms that in all three cases imipramine molecule binds metal cation in an analogous way. In the 1650–1200 cm^−1^ region, several bands correspond to *ν*(CN), *ν*(C=C), *δ*(NH) and *δ*(CH) vibrations, with the sharpest ones: 1594, 1569, 1484, 1478, 1447, 1328, 1329, 1294, 1226 cm^−1^ for imipramine. All of these bands are also present in the studied compounds’ spectra. In the spectra of metal(II) compounds, these bands appear at: [HL]_2_^+^[CuCl_4_]^2−^: 1603, 1556, 1494, 1478, 1447, 1328, 1299, 1245 cm^−1^; [HL]_2_^+^[ZnCl_4_]^2−^: 1592, 1570, 1496, 1473, 1448, 1330, 1306, 1233 cm^−1^; and [HL]_2_^+^[CdCl_4_]^2−^: 1592, 1573, 1482, 1474, 1442, 1330, 1304, 1232 cm^−1^. Moving towards the lower wavelengths, we can observe bands that correspond to γ(CH) modes: [HL]_2_^+^[CuCl_4_]^2−^: 967, 929, 856, 780, 752, 698 cm^−1^; [HL]_2_^+^[ZnCl_4_]^2−^: 973, 914, 881, 758, 754, 710 cm^−1^; and [HL]_2_^+^[CdCl_4_]^2−^: 972, 917, 865, 757, 756, 713 cm^−1^. In the 1650–600 cm^−1^ range, the observed bands clearly overlap, which is an additional confirmation of the isostructurality of described compounds. 

### 2.4. Thermogravimetric Analysis

**Figure 5:**
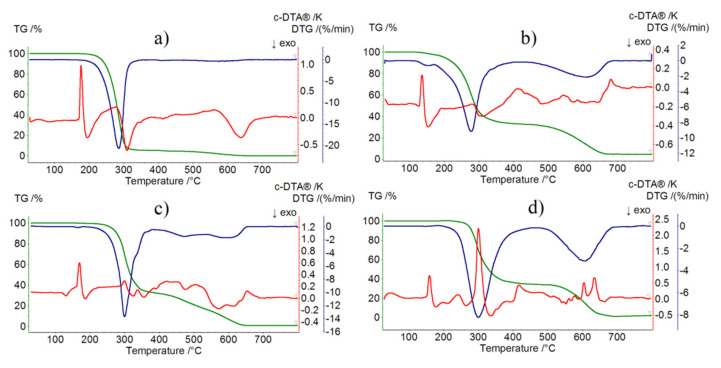
TG, DTG, and DTA curves of: (**a**) imipramine hydrochloride in air, (**b**) [HL]_2_^+^[CuCl_4_]^2−^ compound in air, (**c**) [HL]_2_^+^[ZnCl_4_]^2−^ compound in air, and (**d**) [HL]_2_^+^[CdCl_4_]^2−^ compound in air.

Imipramine hydrochloride itself is stable at up to 175 °C (Figure 5a). At this temperature, it melts; the melting point was determined to be 173.7–174.9 °C, which corresponds very well with the endothermic DTA peak at 175 °C that can be ascribed to this phase change. In the temperature range of 175–340 °C, we observe one major mass loss (95%) (DTG peak at 285 ℃), which is a result of thermal destruction of the compound. Next, from 400 °C up to 660 °C, we observe small mass loss (5%), which can be probably ascribed to post-combustion processes of the organic molecule.

[HL]_2_^+^[CuCl_4_]^2−^ compound begins to decompose at 130 °C (Figure 5b). Its melting point was determined to be 130.3–132.5 °C, which corresponds well with the endothermic DTA peak at 135 °C. Mass loss in the temperature range 130–420 °C is associated with almost total destruction of organic molecules (mass loss found: 68.0%, calc.: 73.27%) (DTG peak at 275 °C). Next, from 460 °C up to 680 °C, we can observe mass loss that corresponds with the destruction of [CuCl_4_]^2−^ (mass loss found: 27.0%, calc.: 26.73%). It is most likely that volatile copper(II) halide derivatives are formed, the existence of which has already been well documented [24].

The determined melting point of [HL]_2_^+^[ZnCl_4_]^2−^ was 165.2–166.6 °C (Figure 5c). Once again, it very well corresponds with the endothermic DTA peak at 170 °C. As well, in this case, the first step of thermal destruction involves almost total decomposition of organic molecules (mass loss found: 68.0%, calc. 73.09%) in the temperature range 170–390 °C. Next, up to 660 °C, we observe total destruction of the molecule (mass loss found: 30.0%, calc. 26.91%).

The [HL]_2_^+^[CdCl_4_]^2−^ compound is stable at up to 220 °C (Figure 5d). The determined melting point (158.2–159.8 °C) corresponds very well with the observed endothermic peak (160 °C). As in all three cases, the first step of decomposition is almost total destruction of organic molecules (mass loss found: 67.0%, calc. 68.89%) that takes place in the temperature range of 220–460 °C. In the temperature range of 460–710 °C, we observe total destruction of the molecule (mass loss found: 33.0%, calc. 31.11%).

### 2.5. Magnetic Studies

All compounds presented a paramagnetic-like behavior, approximately following the Curie–Weiss law:

(1) $$\chi \left(T,H\right)=C\frac{1}{T}+D$$

where ***D*** accounts for a constant diamagnetic background, ***C*** is the Curie constant, and χ≡M/H is the molar magnetic susceptibility.

**Figure 6:**
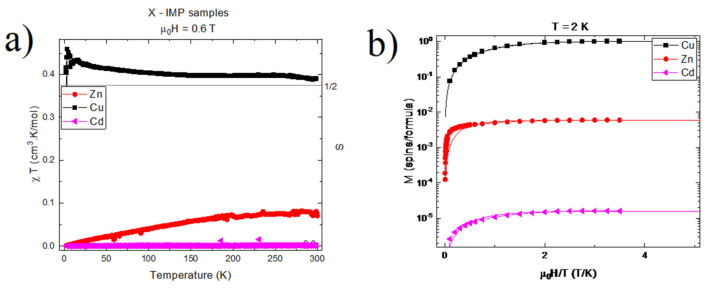
(**a**) Reduced magnetic susceptibility Δχ×T vs. temperature for [HL]_2_[XCl_4_]^2−^, X = Zn, Cu, Cd. The subtracted diamagnetic background was $D=-1.35\times 10^{-2}$$cm^{3}mol^{-1}$ for X = Cu and $D=-6.5\times 10^{-4}$$cm^{3}mol^{-1}$ for X = Zn. No subtraction was performed for X = Cd. Measurements were performed under a magnetic field of $\mu_{0}H=0.6$ T. The horizontal line corresponds to $\Delta \chi T=C\left(J=\frac{1}{2}\right).$ (**b**) Magnetization vs. reduced magnetic field $\mu_{0}H/T$ for [HL]_2_[XCl_4_]^2−^, X = Zn, Cu, Cd. Curves were obtained at T = 2 K, and had their linear diamagnetic contribution removed for clarity. The y-axis is given in units of Bohr magnetons per formula. The solid lines are function of the type $y\propto B_{J}\left(x\right)$, B_J_ the Brillouin function with $x=gJ\mu_{0}\mu_{B}k_{B}^{-1}H/T$. J = 0.59 for Cu, J = 3 for Zn, and J = 1.3 for Cd. g is taken at g = 2.1 for all samples.

The samples’ temperature-varying component of the magnetic susceptibility, χ=χ−D, is presented in Figure 6a through the use of the reduced quantity ΔχT. Results revealed nearly temperature-independent curves above 150 K. For paramagnets, such plots are expected to converge to ΔχT=C. This allows the estimation C and its associated molecular magnetic momentum number ***J***, through

(2) $$C=C\left(J\right)=Ng^{2}\frac{\mu_{0}^{2}\mu_{B}^{2}}{3k_{B}}J\left(J+1\right)$$

In this equation, ***N*** is the Avogadro number, $\mu_{B}$ the Bohr magneton, g the electron’s gyromagnetic factor, and $k_{B}$ the Boltzmann constant. This method yielded J≈1/2 for [HL]_2_^+^[CuCl_4_]^2−^, suggesting a single, non-interacting electronic spin ½ per molecule in this compound. Results for of [HL]_2_^+^[ZnCl_4_]^2−^ and [HL]_2_^+^[CdCl_4_]^2−^, on the other hand, yielded J≈0.08 and $J\approx 2\times 10^{-3}$, respectively, thus suggesting non-magnetic compounds.

Such observations were corroborated through magnetic-field-dependent measurements, as shown in Figure 6b. In this figure, only the paramagnetic component of M(H) is shown. Indeed, M(H) results for [HL]_2_^+^[CuCl_4_]^2−^ presented a paramagnetic contribution of $1\mu_{B}$ per molecule, which was well-described by a Brillouin function B_J_(x), where x is the ratio of the Zeeman to the thermal energy $x=gJ\mu_{0}\mu_{B}k_{B}^{-1}H/T$. The best fit was obtained for ***J*** = 0.5 and ***g*** = 2.1. As in temperature-dependent measurements, these results indicate a single, non-interacting electronic spin 1/2 per molecule in this compound. Conversely, the M(H) responses of [HL]_2_^+^[ZnCl_4_]^2−^ and [HL]_2_^+^[CdCl_4_]^2−^ presented weak paramagnetic contributions, amounting to c.a. $2.5\times 10^{-3}$$\mu_{B}$ and $1\times 10^{-5}$$\mu_{B}$ per molecule, respectively. These values can be attributed either to a small quantity of free radicals, or to the presence of foreign magnetic composites (e.g., magnetite) in quantities as low as 0.2% (impurities/molecule) for the [HL]_2_^+^[ZnCl_4_]^2−^ sample, and 10 ppm for the [HL]_2_^+^[CdCl_4_]^2−^ sample.

### 2.6. Biological Assays

#### 2.6.1. Determination of the Lethal Concentrations for [HL]_2_^+^[CuCl_4_]^2−^ (A), [HL]_2_^+^[ZnCl_4_]^2−^ (B), and [HL]_2_^+^[CdCl_4_]^2−^ (C) in a Solvent-Dependent Manner of Changes (Water, H_2_O/Ethanol, EtOH 0.2%)

**Figure 7:**
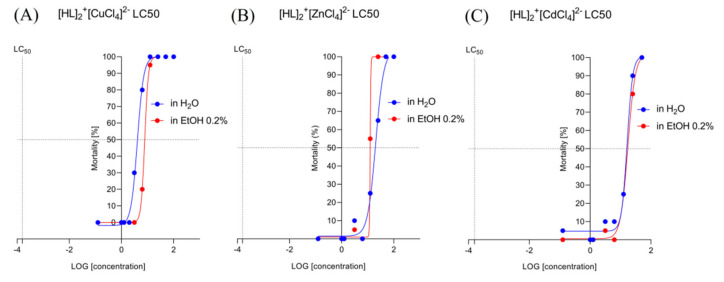
The lethal concentrations (LC50) (log[concentration]) detected for [HL]_2_^+^[CuCl_4_]^2−^ (**A**), [HL]_2_^+^[ZnCl_4_]^2−^ (**B**), and [HL]_2_^+^[CdCl_4_]^2−^ (**C**) in a solvent-dependent manner of changes (water, H_2_O/ethanol, EtOH 0.2%).

Nonlinear fit analysis (Figure 7A) revealed that EC50 for [HL]_2_^+^[CuCl_4_]^2−^ in H_2_O is 4.070 and for EtOH 0.2% 7.803. LogEC50 for [HL]_2_^+^[CuCl_4_]^2−^/H_2_O is 0.6096 and for [HL]_2_^+^[CuCl_4_]^2−^/EtOH is 0.8923. According to the 95% CI (profile likelihood), LogEC50 for [HL]_2_^+^[CuCl_4_]^2−^/H_2_O is situated between 0.5682 to 0.6523, and for [HL]_2_^+^[CuCl_4_]^2−^/EtOH between 0.891 to 0.8954. According to the 95% CI (profile likelihood), EC50 is placed between 3.700 to 4.490 for [HL]_2_^+^[CuCl_4_]^2−^/H_2_O and between 7.746 to 7.859 for [HL]_2_^+^[CuCl_4_]^2−^/EtOH.

Nonlinear fit analysis (Figure 7B) revealed that EC50 for [HL]_2_^+^[ZnCl_4_]^2−^ in H_2_O is 20.25 and for EtOH 0.2% ~ 12.43. LogEC50 for [HL]_2_^+^[ZnCl_4_]^2−^/H_2_O is 1.306 and for [HL]_2_^+^[ZnCl_4_]^2−^/EtOH ~ 1.0957. According to the 95% CI (profile likelihood), LogEC50 for [HL]_2_^+^[ZnCl_4_]^2−^/H_2_O is situated between 1.215 to 1.404. According to the 95% CI (profile likelihood), EC50 is 16.39 to 25.33 for [HL]_2_^+^[ZnCl_4_]^2−^/H_2_O. For [HL]_2_^+^[ZnCl_4_]^2−^/EtOH EC50, Log EC50 was not established by the analysis.

Nonlinear fit analysis (Figure 7C) revealed that EC50 for [HL]_2_^+^[CdCl_4_]^2−^ in H_2_O is 16.37 and for EtOH 0.2% 17.38. LogEC50 for [HL]_2_^+^[CdCl_4_]^2−^/H_2_O is 1.214 and for [HL]_2_^+^[CuCl_4_]^2−^/EtOH, 1.240. According to the 95% CI (profile likelihood), LogEC50 for [HL]_2_^+^[CuCl_4_]^2−^/H_2_O reaches 1298, and for [HL]_2_^+^[CuCl_4_]^2−^/EtOH is between 1.187 to 1.294. According to the 95% CI (profile likelihood), EC50 reaches 19.84 for [HL]_2_^+^[CdCl_4_]^2−^/H_2_O and is between 15.40 to 19.70 for [HL]_2_^+^[CuCl_4_]^2−^/EtOH.

#### 2.6.2. Determination of the Heart Rate of Zebrafish Larvae in 0–5 dpf. The Solvent-Dependent Effect of [HL]_2_^+^[CuCl_4_]^2−^ (A), [HL]_2_^+^[ZnCl_4_]^2−^ (B), and [HL]_2_^+^[CdCl_4_]^2−^ (C) on the Heart Rate of Zebrafish Larvae in 48 hpf (Left) and 96 hpf (Right)

**Figure 8:**
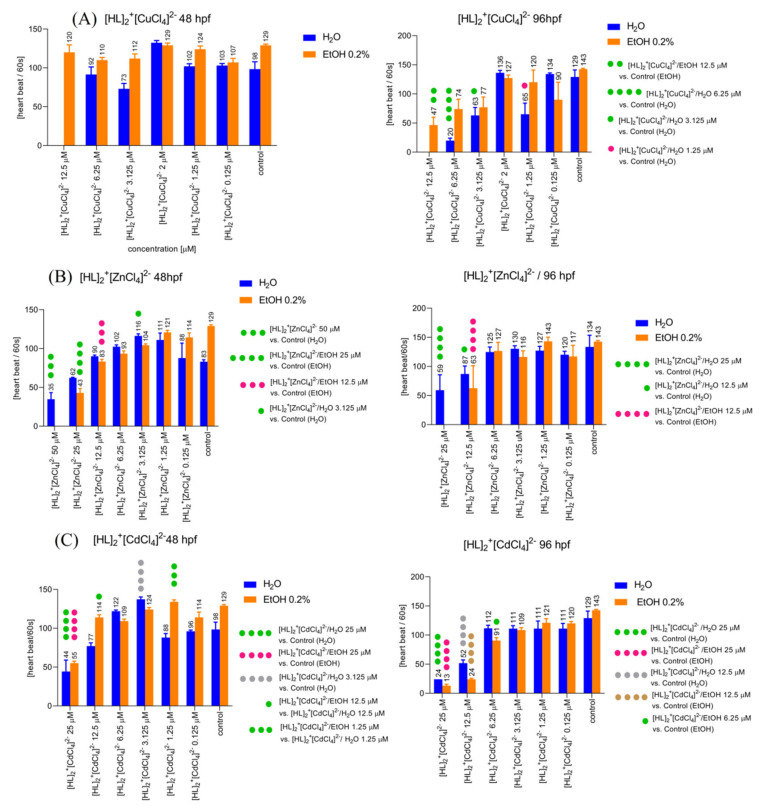
The [HL]_2_^+^[CuCl_4_]^2−^ (**A**), [HL]_2_^+^[ZnCl_4_]^2−^ (**B**), and [HL]_2_^+^[CdCl_4_]^2−^ (**C**) induced influences on the heart rate of Zebrafish larvae in 48 hpf (left) and 96 hpf (right) in the solvent-dependent manner of changes.

Two-way ANOVA analysis showed a statistically significant difference in heart rate after 48 h post fertilization (Figure 8A, left) as a response to the used treatment (F (5, 37) = 13.27, *p* < 0.0001), used solvent (F (1, 37) = 27.09, *p* < 0.0001), drug–solvent interactions (F (5, 37) = 4.376, *p* = 0.0031). The post hoc Bonferroni’s test revealed a significant increase in the heart rate in the group treated with [HL]_2_^+^[CuCl_4_]^2−^ 2 µM compared to the control group, EtOH 0.2% (*p* < 0.001). Two-way ANOVA analysis showed statistically significant difference in heart rate after 96 h post fertilization (Figure 8A, right) as a response to used treatment (F (6, 31) = 27.99, *p* < 0.0001), used solvent (F (1, 31) = 8.409, *p* = 0.0068), drug–solvent interactions (F (6, 31) = 4.502, *p* = 0.0022). The post hoc Bonferroni’s test revealed a significant decrease in the heart rate in the group treated with [HL]_2_^+^[CuCl_4_]^2−^/EtOH 12.5 µM compared to the control group, EtOH 0.2% (*p* < 0.01), significant decrease in the heart rate after treatment with [HL]_2_^+^[CuCl_4_]^2−^/H_2_O 6.25 µM (*p* < 0.001), [HL]_2_^+^[CuCl_4_]^2−^/H_2_O 3.125 µM (*p* = 0.05), and [HL]_2_^+^[CuCl_4_]^2−^/H_2_O 1.25 µM (*p* < 0.05) when compared to control group (E3).

Two-way ANOVA analysis showed statistically significant difference in heart rate after 48 h post fertilization (Figure 8B, left) as a response to used treatment (F (7, 85) = 86.91, *p* < 0.0001), drug–solvent interactions (F (7, 85) = 9.487, *p* < 0.0001) and non-significant difference in response to the treatment with applied solvents (F (1, 85) = 0.0004440, *p* = 0.9832). The post hoc Bonferroni’s test revealed a significant decrease in the heart rate in the group treated with [HL]_2_^+^[ZnCl_4_]^2−^/H_2_O 50 µM (*p* < 0.001), and significant increase in a heart rate after application of [HL]_2_^+^[ZnCl_4_]^2−^/H_2_O 3.125 µM (*p* < 0.05) compared to the control group (E3). Additionally, the post hoc Bonferroni’s test revealed a significant decrease in a heart rate after treatment with [HL]_2_^+^[ZnCl_4_]^2−^/EtOH 25 µM (*p* < 0.0001) and 12 µM (*p* < 0.001) when compared with the control group (EtOH 0.2%). Two-way ANOVA analysis showed a statistically significant difference in heart rate after 96 h post fertilization (Figure 8B, right) as a response to used treatment (F (6, 63) = 48.30, *p* < 0.0001), used solvent (F (1, 63) = 5.358, *p* = 0.0239), and drug–solvent interactions (F (6, 63) = 4.812, *p* = 0.0004). The post hoc Bonferroni’s test revealed a significant decrease in the heart rate in the group treated with [HL]_2_^+^[ZnCl_4_]^2−^/H_2_O 25 µM (*p* < 0.0001), and [HL]_2_^+^[ZnCl_4_]^2−^/H_2_O 12.5 µM (*p* < 0.05) when compared to the control group (E3). Additionally, the post hoc Bonferroni’s test revealed a significant decrease in a heart rate after treatment with [HL]_2_^+^[ZnCl_4_]^2−^/EtOH 12.5 µM (*p* < 0.0001) when compared with the control group (EtOH 0.2%).

Two-way ANOVA analysis showed statistically significant difference in heart rate after 48 h post fertilization (Figure 8C, left) as a response to used treatment (F (6, 51) = 42.20, *p* < 0.0001), used solvent (F (1, 51) = 28.14, *p* < 0.0001), and drug–solvent interactions (F (6, 51) = 10.07, *p* < 0.0001). The post hoc Bonferroni’s test revealed a significant decrease in the heart rate in the group treated with [HL]_2_^+^[CdCl_4_]^2−^/H_2_O 25 µM (*p* < 0.0001), and [HL]_2_^+^[CdCl_4_]^2−^/EtOH 25 µM (*p* < 0.0001) when compared with the control groups E3 and EtOH 0.2%, respectively. However, the treatment with [HL]_2_^+^[CdCl_4_]^2−^/H_2_O 3.125 µM (*p* < 0.0001) caused an increase in the heart rate when compared with the control group (E3). Moreover, there was a significant difference in the heart rate between [HL]_2_^+^[CdCl_4_]^2−^/H_2_O 12.5 and [HL]_2_^+^[CdCl_4_]^2−^/EtOH 12.5 (*p* < 0.05), as well as between [HL]_2_^+^[CdCl_4_]^2−^/H_2_O 1.25 and [HL]_2_^+^[CdCl_4_]^2−^/EtOH 1.25 (*p* < 0.001). Two-way ANOVA analysis showed non-significant difference in heart rate after 96 h post fertilization (Figure 8C, right) in response to the treatment (F (6, 37) = 46.19, *p* < 0.0001), and drug–solvent interactions (F (6, 37) = 2.224, *p* = 0.0624), and significant difference in response to the treatment with applied solvents (F (1, 37) = 0.9210, *p* = 0.3435). The post hoc Bonferroni’s test revealed a significant decrease in the heart rate in the group treated with [HL]_2_^+^[CdCl_4_]^2−^/H_2_O 25 µM (*p* < 0.0001), and [HL]_2_^+^[CdCl_4_]^2−^/EtOH 25 µM (*p* < 0.0001) when compared to the control groups E3 and EtOH 0.2%, respectively. Moreover, the post hoc Bonferroni’s test showed a significant decrease in a heart rate after treatment with [HL]_2_^+^[CdCl_4_]^2−^/H_2_O 12.5 µM (*p* < 0.0001), and [HL]_2_^+^[CdCl_4_]^2−^/EtOH 12.5 µM (*p* < 0.0001) when compared with the control groups E3 and EtOH 0.2%, respectively. Additionally, [HL]_2_^+^[CdCl_4_]^2−^/EtOH 6.25 µM caused a significant decrease in the heart rate when compared with the control (EtOH 0.2%, *p* < 0.05).

#### 2.6.3. Determination of the Hatching Rate of Zebrafish Larvae in 0–5 dpf. Solvent-Dependent (Water (Left)/Ethanol (Right)) Effect of [HL]_2_^+^[CuCl_4_]^2−^ (A), [HL]_2_^+^[ZnCl_4_]^2−^ (B), and [HL]_2_^+^[CdCl_4_]^2−^ (C), Manner of Changes in the Hatching Rate after 48 hpf, 72 hpf, and 96 hpf of Zebrafish Larvae

**Figure 9:**
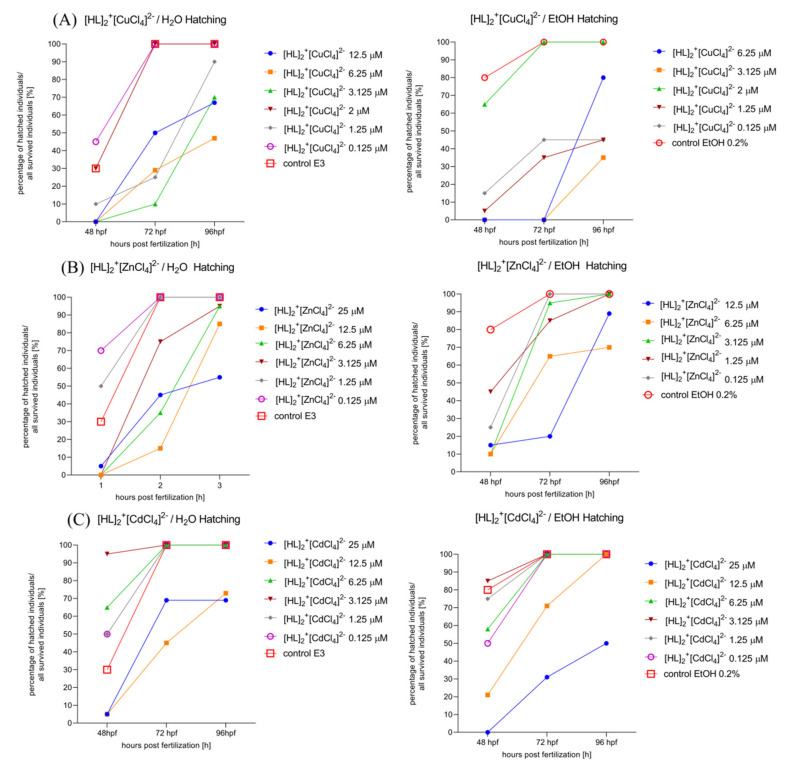
Solvent-dependent effect (ethanol (**left**)/water (**right**)) effect of [HL]_2_^+^[CuCl_4_]^2−^ (**A**), [HL]_2_^+^[ZnCl_4_]^2−^ (**B**), and [HL]_2_^+^[CdCl_4_]^2−^ (**C**) on the hatching rate after 48 hpf, 72 hpf, and 96 hpf of Zebrafish larvae.

One-way ANOVA analysis showed a non-significant difference in hatching in response to the tested compounds dissolved initially in H_2_O (Figure 9A, left) (F (1.232, 2.464) = 7.265, *p* = 0.0922), and significant changes in hatching rate after 48, 72, and 96 h post fertilization (F (2, 12) = 30.26, *p* < 0.0001). Moreover, one-way ANOVA analysis showed a non-significant difference in hatching in response to the tested compounds dissolved primarily in EtOH (Figure 9A, right) (F (1.183, 2.366) = 12.74, *p* = 0.0546) and significant changes in hatching rate after rate after 48, 72 and 96 h post fertilization (F (2, 10) = 8.484, *p* = 0.0070).

One-way ANOVA analysis showed a non-significant difference in hatching in response to the tested compounds dissolved initially in H_2_O (Figure 9B, left) (F (1.760, 3.520) = 5.026, *p* = 0.0940, *p* = 0.0940), and significant changes in hatching rate after 48, 72, and 96 h post fertilization (F (2, 12) = 25.06, *p* < 0.0001). Moreover, one-way ANOVA analysis showed non-significant difference in hatching in response to the tested compounds dissolved primarily in EtOH (Figure 9B, right) (F (1.836, 3.672) = 2.960, *p* = 0.1714) and significant changes in hatching rate after rate after 48, 72, and 96 h post fertilization (F (2, 10) = 16.73, *p* = 0.0006).

One-way ANOVA analysis showed a significant difference in hatching rate in response to the tested compounds dissolved initially in H_2_O (Figure 9C, left) (F (1.255, 2.510) = 12.77, *p* = 0.0493), and significant changes in hatching rate after 48, 72, and 96 h post fertilization (F (2, 12) = 28.12, *p* < 0.0001). Moreover, one-way ANOVA analysis showed significant difference in hatching rate in response to the tested compounds dissolved primarily in EtOH (Figure 9C, right) (F (1.255, 2.510) = 12.77, *p* = 0.0493) and significant changes in hatching rate after 48, 72, and 96 h post fertilization (F (2, 12) = 22.93, *p* < 0.0001).

#### 2.6.4. [HL]_2_^+^[CuCl_4_]^2−^ (A), [HL]_2_^+^[ZnCl_4_]^2−^ (B), and [HL]_2_^+^[CdCl_4_]^2−^ (C) Induce Zebrafish Larvae’s Development Malformations in the FET Test (0–5 dpf)

Figures showing induced Zebrafish larvae’s development malformations can be found in the Supplementary Material (Figures S1–S9).

#### 2.6.5. Assessment of the Mortality Rate within 96 h in Order to Choose the Safest Doses for Behavioral Study. [HL]_2_^+^[CuCl_4_]^2−^ (A), [HL]_2_^+^[ZnCl_4_]^2−^ (B), and [HL]_2_^+^[CdCl_4_]^2−^ (C) Solvent-Dependent (H_2_O, Left; EtOH 0.2%, right) Influence on the Zebrafish Larvae’s Mortality Rate

**Figure 10:**
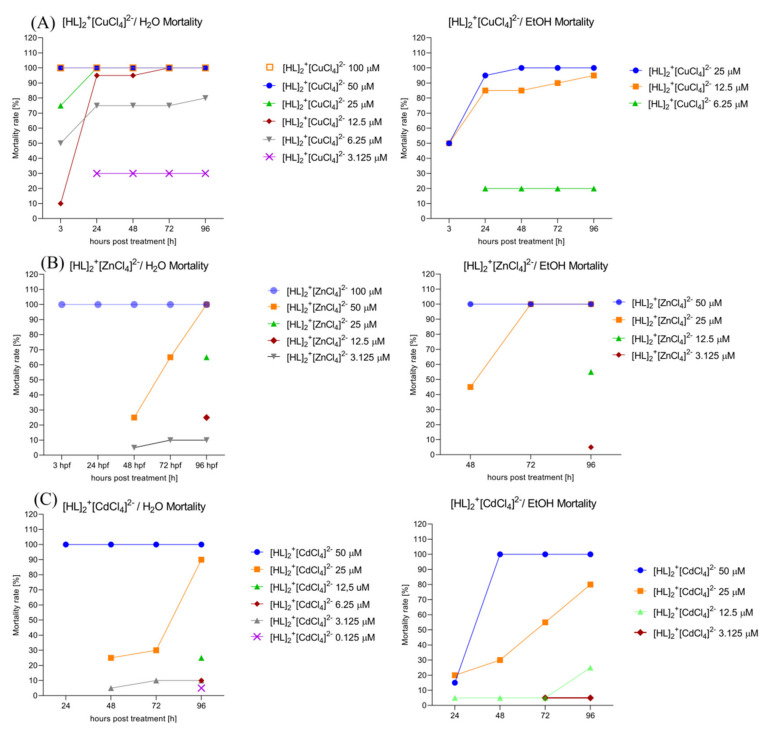
The [HL]_2_^+^[CuCl_4_]^2−^ (**A**), [HL]_2_^+^[ZnCl_4_]^2−^ (**B**), and [HL]_2_^+^[CdCl_4_]^2−^ (**C**) influence on the solvent-dependent (H_2_O (**left**); EtOH 0.2% (**right**)) Zebrafish larvae’s mortality rate.

Two-way ANOVA analysis revealed a significant difference in mortality rate (%) depending on the treatment with the drug dissolved initially in the water (Figure 10A, left) (F (4, 28) = 3.933, *p* = 0.0117) and hours of exposure to the treatment (F (7, 28) = 52.83, *p* < 0.0001). Bonferroni’s post hoc test showed the same significant decrease in mortality after 3, 24, 48, 72, and 96 h of treatment with [HL]_2_^+^[CuCl_4_]^2−^ 100 μM, 50 μM, and 25 μM in comparison with [HL]_2_^+^[CuCl_4_]^2−^ 3.125 μM, 1.25 μM, 0.125 μM, and the control group (E3) (*p* < 0.0001), and significant increase in mortality rate caused by [HL]_2_^+^[CuCl_4_]^2−^ 100 μM, 50 μM, 25 μM, 12.5 μM, and 6.25 μM in comparison to the control group (E3) (*p* < 0.0001). There was no significant difference in mortality rate between [HL]_2_^+^[CuCl_4_]^2−^ 3.125 μM, 1.25 μM, 0.125 μM, and the control group (E3). Two-way ANOVA analysis revealed a significant difference in mortality rate (%) depending on the treatment with the drug dissolved initially in ethanol (Figure 10A, right) (F (4, 20) = 4.008, *p* = 0.0151) and hours of exposure to the drug (F (5, 20) = 92.10, *p* < 0.0001). Bonferroni’s post hoc test showed the same significant decrease in mortality rate after 3, 24, 48, 72, and 96 h of exposure to the [HL]_2_^+^[CuCl_4_]^2−^ 25 μM/12.5 µM when compared to [HL]_2_^+^[CuCl_4_]^2−^ 6.25 μM, 3.125 μM, 1.25 μM, 0.125 μM, and control EtOH 0.2% (*p* < 0.0001). There was no significant changes in mortality rate between groups after the treatment with [HL]_2_^+^[CuCl_4_]^2−^ 6.26 μM, 3.125 μM, 1.25 μM, 0.125 μM, and the control (EtOH 0.2%).

Two-way ANOVA analysis revealed a significant difference in mortality rate (%) depending on the treatment with the drug dissolved initially in the water (Figure 10B, left) (F (8, 32) = 20.65, *p* < 0.0001) and hours of exposure to the treatment (F (4, 32) = 2.917, *p* = 0.0365). Bonferroni’s post hoc test showed a significant decrease in mortality after 3, 24, 48, 72, and 96 h of treatment between [HL]_2_^+^[ZnCl_4_]^2−^ 100 μM and [HL]_2_^+^[ZnCl_4_]^2−^ 50 μM (*p* < 0.01), 25 μM, 12.5 μM, 6.25 μM, 3.125 μM, 1.25 μM, 0.125 μM and the control group E3 (*p* < 0.0001). There was no significant difference in mortality rate between [HL]_2_^+^[CuCl_4_]^2−^ 3.125 μM, 1.25 μM, 0.125 μM, and the control group (E3). Two-way ANOVA analysis revealed a significant difference in mortality rate (%) depending on the treatment with the drug dissolved initially in ethanol (Figure 10B, right) (F (7, 28) = 4.891, *p* = 0.0011) and hours of exposure to the drug (F (4, 28) = 2.821, *p* = 0.0439). Bonferroni’s post hoc test showed no significant changes between groups in the multiple comparison method.

Two-way ANOVA analysis revealed a significant difference in mortality rate (%) depending on the treatment with the drug dissolved initially in water (Figure 10C, left) (F (4, 28) = 2.495, *p* = 0.0656) and hours of exposure to the drug (F (7, 28) = 10.44, *p* < 0.0001). Bonferroni’s post hoc test showed the same significant decrease in mortality rate after 3, 24, 48, 72, and 96 h of exposure to the [HL]_2_^+^[CdCl_4_]^2−^ 50 μM when compared to [HL]_2_^+^[CdCl_4_]^2−^ 12.5 μM, 6.25 μM, 3.125 μM, 1.25 μM, 0.125 μM, and control EtOH 0.2% (*p* < 0.001). There were no significant changes between groups after the treatment with [HL]_2_^+^[CdCl_4_]^2−^ 25 μM, 12.5 μM, 6.26 μM, 3.125 μM, 1.25 μM, 0.125 μM, and the control (E3). Two-way ANOVA analysis revealed a significant difference in mortality rate (%) depending on the treatment with the drug dissolved initially in the ethanol (Figure 10C, right) (F (4, 28) = 2.474, *p* = 0.0673) and hours of exposure to the treatment (F (7, 28) = 7.184, *p* < 0.0001). Bonferroni’s post hoc test showed the same significant decrease in mortality after 3, 24, 48, 72, and 96 h of treatment with [HL]_2_^+^[CdCl_4_]^2−^ 50 μM when compared to [HL]_2_^+^[CdCl_4_]^2−^ 6.25 μM, 3.125 μM, 1.25 μM, 0.125 μM, and control EtOH 0.2% (*p* < 0.05). There was no significant difference in mortality rate between [HL]_2_^+^[CdCl_4_]^2−^ 25 μM, 12.5 μM, 6.25 μM, 3.125 μM, 1.25 μM, 0.125 μM, and the control group (E3).

#### 2.6.6. [HL]_2_^+^[CuCl_4_]^2^ (A), [HL]_2_^+^[ZnCl_4_]^2−^ (B), and [HL]_2_^+^[CdCl_4_]^2−^ (C) Influence on the Spontaneous Locomotor Activity When Compared to Diazepam and Imipramine. Solvent-Dependent Effect Evaluation

**Figure 11:**
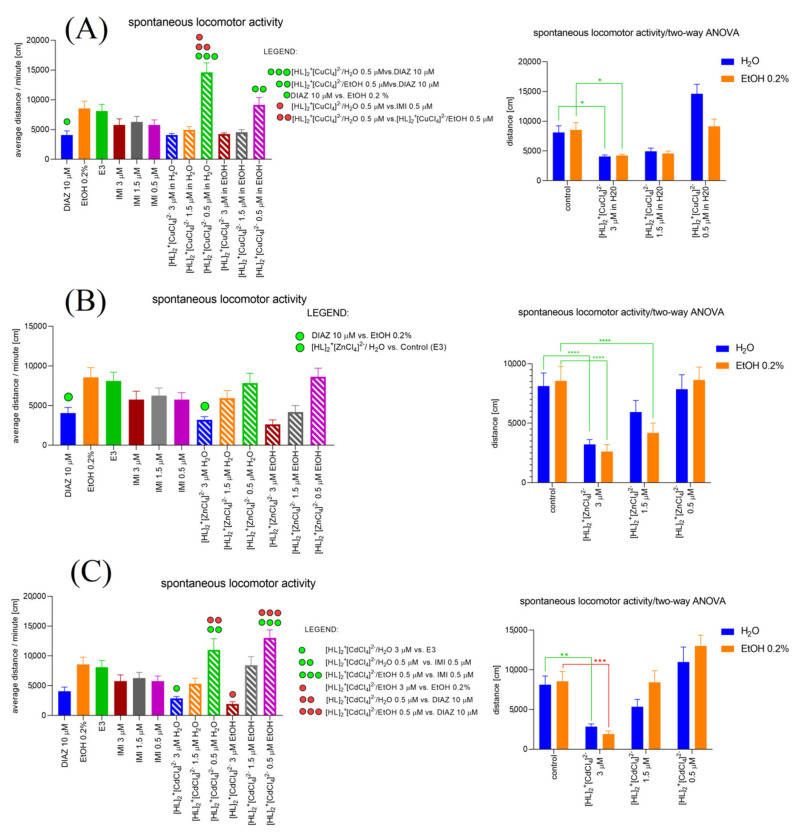
[HL]_2_^+^[CuCl_4_]^2^ (**A**), [HL]_2_^+^[ZnCl_4_]^2−^ (**B**), and [HL]_2_^+^[CdCl_4_]^2−^ (**C**) influence on the Zebrafish larvae’s spontaneous locomotor activity compared with the changes caused by imipramine and diazepam (**left**). Evaluation of the solvent-dependent manner of changes in the spontaneous locomotor activity of Zebrafish larvae under the influence of [HL]_2_^+^[CuCl_4_]^2^ (**A**), [HL]_2_^+^[ZnCl_4_]^2−^ (**B**), and [HL]_2_^+^[CdCl_4_]^2−^ (**C**) (**right**).

One-way ANOVA analysis (Figure 11A) revealed a significant difference in the spontaneous locomotor activity (average distance/minute (cm)) caused by treatment (F (11, 276) = 10.69, *p* < 0.0001). Tukey’s post hoc test revealed significant increase in the average distance/minute (cm) after [HL]_2_^+^[CuCl_4_]^2−^/H_2_O 0.5 µM (*p* < 0.0001) and [HL]_2_^+^[CuCl_4_]^2−^/EtOH 0.5 µM (*p* < 0.01) treatment when compared with DIAZ 10 µM. Moreover, Tukey’s post hoc test showed significant increase in the average distance/minute (cm) after [HL]_2_^+^[CuCl_4_]^2−^/H_2_O 0.5 µM when compared with IMI 0.5 µM (*p* < 0.0001). When the treatment with [HL]_2_^+^[CuCl_4_]^2−^/H_2_O 0.5 µM was applied the average distance/minute (cm) was significantly increased when compared with [HL]_2_^+^[CuCl_4_]^2−^/EtOH 0.5 µM (*p* < 0.01). Two-way ANOVA analysis revealed significant difference in spontaneous locomotor activity (average distance/minute (cm)) in a treatment (F (2.064, 47.47) = 21.38, *p* < 0.0001), used solvent (F (1, 23) = 4.764, *p* = 0.0395), and solvent–treatment interaction (F (1.409, 32.40) = 8.278, *p* = 0.0033). Bonferroni’s post hoc test revealed a significant decrease in the spontaneous locomotor activity after [HL]_2_^+^[CuCl_4_]^2−^/H_2_O 3 µM and [HL]_2_^+^[CuCl_4_]^2−^/EtOH 3 µM treatment when compared to the control groups (*p* < 0.05).

One-way ANOVA analysis (Figure 11B) revealed a significant difference in the spontaneous locomotor activity (average distance/minute (cm)) caused by treatment (F (11, 252) = 4.249, *p* < 0.0001). Tukey’s post hock test revealed significant decrease in the average distance/minute (cm) after [HL]_2_^+^[ZnCl_4_]^2−^/H_2_O 3 µM (*p* < 0.05) treatment when compared with the control group, E3. Two-way ANOVA analysis revealed significant difference in spontaneous locomotor activity (average distance/minute (cm)) in a treatment (F (1, 23) = 23.66, *p* < 0.0001), used solvent (F (3, 69) = 38.29, *p* < 0.0001) and solvent–treatment interaction (F (3, 45) = 5.606, *p* = 0.0024). Bonferroni’s post hoc test revealed significant decrease in the spontaneous locomotor activity after [HL]_2_^+^[ZnCl_4_]^2−^/H_2_O 3 µM and [HL]_2_^+^[ZnCl_4_]^2−^/EtOH 3 µM treatment when compared to the control groups, E3 and EtOH 0.2%, respectively (*p* < 0.0001). Moreover, Bonferroni’s post hoc test showed a significant decrease in spontaneous locomotor activity after [HL]_2_^+^[ZnCl_4_]^2−^/EtOH 1.5 µM treatment when compared to the control group (EtOH 0.2%) (*p* < 0.0001).

One-way ANOVA analysis (Figure 11C) revealed a significant difference in the spontaneous locomotor activity (average distance/minute (cm)) caused by treatment (F (11, 276) = 8.648, *p* < 0.0001). Tukey’s post hoc test revealed significant decrease in the average distance/minute (cm) after [HL]_2_^+^[CdCl_4_]^2−^/H_2_O 3 µM (*p* < 0.05) and [HL]_2_^+^[CdCl_4_]^2−^/EtOH 3 µM (*p* < 0.01) treatment when compared with the control groups, E3 and EtOH 0.2%, respectively. Moreover, Tukey’s post hoc test showed significant increase in the average distance/minute (cm) after [HL]_2_^+^[CdCl_4_]^2−^/H_2_O 0.5 µM when compared with IMI 0.5 µM (*p* < 0.05), and DIAZ 10 µM (*p* < 0.001). When the treatment with [HL]_2_^+^[CdCl_4_]^2−^/EtOH 0.5 µM was applied the average distance/minute (cm) was significantly increased when compared with IMI 0.5 µM (*p* < 0.001) and DIAZ 10 µM (*p* < 0.0001). Two-way ANOVA analysis revealed no significant difference in spontaneous locomotor activity (average distance/minute (cm)) in a treatment (F (1, 23) = 1.811, *p* = 0.1915) and solvent–treatment interaction (F (3, 69) = 1.602, *p* = 0.1969), and significant difference in the spontaneous locomotor activity after solvent influence (F (3, 69) = 28.53, *p* < 0.0001). Bonferroni’s post hoc test revealed significant decrease in the spontaneous locomotor activity after [HL]_2_^+^[CdCl_4_]^2−^/H_2_O 3 µM (*p* < 0.01) and [HL]_2_^+^[CdCl_4_]^2−^/EtOH 3 µM (*p* < 0.001) treatment when compared to the control groups, E3 and EtOH 0.2%, respectively.

#### 2.6.7. [HL]_2_^+^[CuCl_4_]^2−^ (A), [HL]_2_^+^[ZnCl_4_]^2−^ (B), and [HL]_2_^+^[CdCl_4_]^2−^ (C) Influences the Zebrafish Larvae’s Light–Dark Stimulated Locomotor Activity (cm) in a Solvent-Dependent Manner

Figures showing [HL]_2_^+^[CuCl_4_]^2−^ (A), [HL]_2_^+^[ZnCl_4_]^2−^ (B), and [HL]_2_^+^[CdCl_4_]^2−^ (C) influence on the Zebrafish larvae’s light–dark affected locomotor activity (cm) in a solvent-dependent manner of changes can be found in the Supplementary Material (Figure S10).

Two-way ANOVA analysis showed significant changes in the average distance/minute (cm) determined by light and dark phases as a response to the treatment (F (1, 71) = 13.28, *p* = 0.0005), the phase (light/dark) (F (11, 781) = 6.945, *p* < 0.0001), and treatment–phase (light/dark) interaction (F (11, 781) = 7.818, *p* < 0.0001). Bonferroni’s post hoc test revealed the significant increase in the average distance/minute (cm) in the dark phase after the treatment with [HL]_2_^+^[CuCl_4_]^2−^/EtOH 1.5 µM when compared to [HL]_2_^+^[CuCl_4_]^2−^/H_2_O 1.5 µM (the difference caused by the solvent) (*p* < 0.001), DIAZ 10 µM (*p* < 0.001), and IMI 1.5 µM (*p* < 0.01). Moreover, the test revealed significant increase in the average distance/minute (cm) caused by the dark phase after the treatment with [HL]_2_^+^[CuCl_4_]^2−^/EtOH 1.5 µM when compared with the light phase (*p* < 0.0001). Additionally, in the dark phase, there was more significant increase in the average distance/minute (cm) after applying [HL]_2_^+^[CuCl_4_]^2−^/EtOH 0.5 µM when compared to [HL]_2_^+^[CuCl_4_]^2−^/H_2_O 0.5 µM (solvent dependent changes), DIAZ 10 µM (*p* < 0.0001), IMI 0.5 µM (*p* < 0.0001), and the control group (EtOH 0.2%) (*p* < 0.0001).

Two-way ANOVA analysis showed significant changes in the average distance/minute (cm) determined by light and dark phases as a response to the treatment (F (1, 1560) = 115.6, *p* < 0.0001), the phase (light/dark) (F (11, 1560) = 26.19, *p* < 0.0001), and treatment–phase (light/dark) interaction (F (11, 1560) = 5.545, *p* < 0.0001). Bonferroni’s post hoc test revealed the significant decrease in the average distance/minute (cm) in the light phase after [HL]_2_^+^[ZnCl_4_]^2−^/H_2_O 3 µM (*p* < 0.01), and [HL]_2_^+^[ZnCl_4_]^2−^/H_2_O 3 (*p* < 0.05)/ [HL]_2_^+^[ZnCl_4_]^2−^/EtOH 1.5 (*p* < 0.0001) when compared with the control group, E3 and EtOH 0.2%, respectively. Moreover, in the dark phase, there was also a significant decrease after [HL]_2_^+^[ZnCl_4_]^2−^/H_2_O 3 µM (*p* < 0.0001) and [HL]_2_^+^[ZnCl_4_]^2−^/EtOH 3 µM (*p* < 0.0001) when compared to the control groups, E3 and EtOH, respectively. In the light phase, there was a significant increase after [HL]_2_^+^[ZnCl_4_]^2−^/H_2_O 1.5 µM (*p* < 0.05) and [HL]_2_^+^[CdCl_4_]^2−^/H_2_O 0.5 µM (*p* < 0.001) when compared to [HL]_2_^+^[CdCl_4_]^2−^/EtOH 1.5 µM and [HL]_2_^+^[CdCl_4_]^2−^/EtOH 0.5 µM, respectively. However, in the dark phase, there was a significant increase after [HL]_2_^+^[ZnCl_4_]^2−^/H_2_O 1.5 μM (*p* < 0.0001), [HL]_2_^+^[ZnCl_4_]^2−^/H_2_O 0.5 μM (*p* < 0.0001), and [HL]_2_^+^[ZnCl_4_]^2−^/EtOH 0.5 μM (*p* < 0.0001) and in the light phase after [HL]_2_^+^[ZnCl_4_]^2−^/H_2_O 0.5 μM (*p* < 0.0001) when compared with DIAZ 10 µM in the dark and light phases, respectively. Moreover, there was a significant decrease after [HL]_2_^+^[ZnCl_4_]^2−^/H_2_O/ light 0.5 μM when compared to [HL]_2_^+^[ZnCl_4_]^2−^/H_2_O/dark 0.5 μM (*p* < 0.01), and significant decrease after [HL]_2_^+^[ZnCl_4_]^2−^/EtOH/light 1.5 μM (*p* < 0.0001) and [HL]_2_^+^[ZnCl_4_]^2−^/EtOH/light 0.5 μM (*p* < 0.0001) when compared to [HL]_2_^+^[ZnCl_4_]^2−^/EtOH/dark 1.5 μM and [HL]_2_^+^[ZnCl_4_]^2−^/EtOH/dark 0.5 μM, respectively. Moreover, the Bonferroni’s post hoc test revealed a significant increase after [HL]_2_^+^[ZnCl_4_]^2−^/H_2_O in the light phase 0.5 μM (*p* < 0.01), and [HL]_2_^+^[ZnCl_4_]^2−^/H_2_O 0.5 μM (*p* < 0.0001), [HL]_2_^+^[ZnCl_4_]^2−^/EtOH 0.5 μM (*p* < 0.0001) in the dark phase when compared to IMI 0.5 μM in the light and IMI 0.5 μM in the dark phase, respectively. However, there was a significant decrease in the dark phase after [HL]_2_^+^[ZnCl_4_]^2−^/EtOH 3 μM application (*p* < 0.05) when compared with IMI 3 μM.

Two-way ANOVA analysis showed significant changes in the average distance/minute (cm) determined by light and dark phases as a response to the treatment (F (1, 1704) = 72.55, *p* < 0.0001), the phase (light/dark) (F (11, 1704) = 41.45, *p* < 0.0001), and treatment–phase (light/dark) interaction (F (11, 1704) = 2.781, *p* = 0.0014). Bonferroni’s post hoc test revealed the significant decrease in the average distance/minute (cm) in the light phase [HL]_2_^+^[CdCl_4_]^2−^/H_2_O 3 µM and [HL]_2_^+^[CdCl_4_]^2−^/EtOH 3 µM when compared with the control groups E3 (*p* < 0.01) and EtOH (0.2%) (*p* < 0.0001), respectively. In the dark phase, the difference was also significant after [HL]_2_^+^[CdCl_4_]^2−^/H_2_O 3 µM and [HL]_2_^+^[CdCl_4_]^2−^/EtOH 3 µM when compared with the control groups, E3 (*p* < 0.0001) and EtOH 0.2% (*p* < 0.0001), respectively. Moreover, there was significant decrease in the average distance/minute (cm) in the light phase after the treatment with [HL]_2_^+^[CdCl_4_]^2−^/H_2_O 1.5 µM and [HL]_2_^+^[CdCl_4_]^2−^/EtOH 1.5 µM when compared with the control groups, E3 (*p* < 0.01) and EtOH 0.2% (*p* < 0.0001), respectively. In contrast, in the dark phase, there was significant increase in the average distance/minute (cm) after [HL]_2_^+^[CdCl_4_]^2−^/H_2_O 0.5 µM when compared with the control group, E3 (*p* < 0.05). Moreover, in the light phase, there was a significant decrease after [HL]_2_^+^[CdCl_4_]^2−^/EtOH 1.5 µM (*p* < 0.01) when compared with IMI 1.5 µM, and significant increase in the dark after [HL]_2_^+^[CdCl_4_]^2−^/EtOH 1.5 µM when compared with DIAZ 10 µM (*p* < 0.05). After the treatment with [HL]_2_^+^[CdCl_4_]^2−^/H_2_O 3 µM, there was a significant decrease in the average distance/minute (cm) in the dark phase (*p* < 0.001), whereas after [HL]_2_^+^[CdCl_4_]^2−^/EtOH 3 µM in the light phase (*p* < 0.001), when compared with the IMI 3 µM dark and light phase, respectively. After the treatment with [HL]_2_^+^[CdCl_4_]^2−^/H_2_O 0.5 µM, there was a significant increase (*p* < 0.001) in the light, (*p* < 0.0001) in the dark phase and [HL]_2_^+^[CdCl_4_]^2−^/EtOH 0.5 µM in the light (*p* < 0.05), in the dark (*p* < 0.0001) when compared to IMI 0.5 µM in the light and dark phases, respectively. Moreover, there was the same significant increase after the treatment with [HL]_2_^+^[CdCl_4_]^2−^/H_2_O 0.5 in the light (*p* < 0.0001), in the dark (*p* < 0.0001) and [HL]_2_^+^[CdCl_4_]^2−^/EtOH 0.5 in the light (*p* < 0.0001), in the dark (*p* < 0.0001) when compared to DIAZ 10 µM in the light and dark phases, respectively.

#### 2.6.8. The Changes Detected during 45 Min of the Locomotor Activity Test. Light-Dark-Dependent Activity. [HL]_2_^+^[CuCl_4_]^2−^, [HL]_2_^+^[ZnCl_4_]^2−^, and [HL]_2_^+^[CdCl_4_]^2−^ Influence on The Light–Dark Dependent Locomotor Activity in a Solvent-Dependent Manner

**Figure 12:**
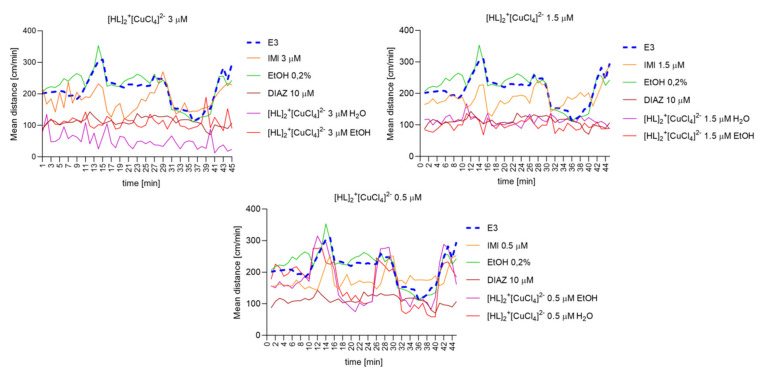
The changes detected during 45 min of the locomotor activity test (three 15-min light–dark sessions: 0–10-min of illumination followed by 11–15 min of darkness, 16–25 min of illumination followed by 26–30 min of darkness, and 31–40 min of illumination followed by 41–45 min of darkness) after 40 min of continuous illumination) under the influence of different concentrations of [HL]_2_^+^[CuCl_4_]^2−^ in the solvent-dependent and light–dark-dependent manner of changes.

Repeated measure one-way ANOVA analysis (Figure 12) revealed significant difference in mean distance cm/min as a result of a treatment: 3 µM: (F (2.464, 108.4) = 201.6, *p* < 0.0001), and the time (F (44, 220) = 2.595, *p* < 0.0001); 1.5 µM: (F (2.037, 89.63) = 152.2, *p* < 0.0001), and the time (F (44, 220) = 2.616, *p* < 0.0001); 0.5 µM: (F (2.638, 116.1) = 45.74, *p* < 0.0001), and the time (F (44, 220) = 5.658, *p* < 0.0001).

**Figure 13:**
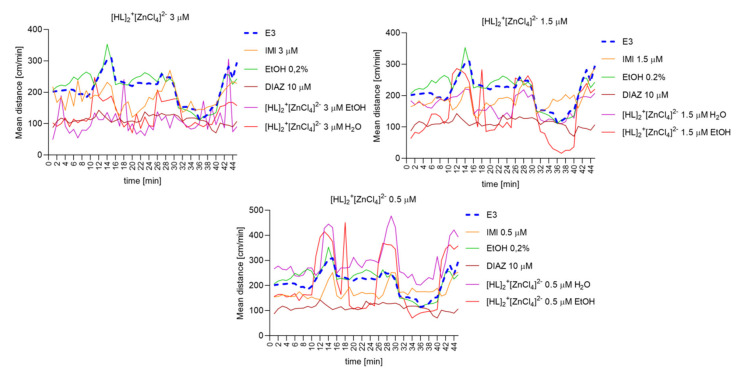
The changes detected during 45 min of the locomotor activity test (three 15-min light–dark sessions: 0–10-min of illumination followed by 11–15 min of darkness, 16–25 min of illumination followed by 26–30 min of darkness, and 31–40 min of illumination followed by 41–45 min of darkness) after 40 min of continuous illumination) under the influence of different concentrations of [HL]_2_^+^[ZnCl_4_]^2−^ in the solvent-dependent and light–dark-dependent manner of changes.

Repeated measure one-way ANOVA analysis (Figure 13) revealed significant difference in mean distance cm/min as a result of a treatment: 3 µM: (F (3.016, 132.7) = 94.75, *p* < 0.0001), and the time (F (44, 220) = 3.702, *p* < 0.0001).; 1.5 µM: (F (2.255, 99.22) = 55,74, *p* < 0,0001), and the time (F (44, 220) = 5.762, *p* < 0.0001); 0.5 µM: (F (2.056, 90.45) = 64.52, *p* < 0.0001), and the time (F (44, 220) = 5.068, *p* < 0.0001).

**Figure 14:**
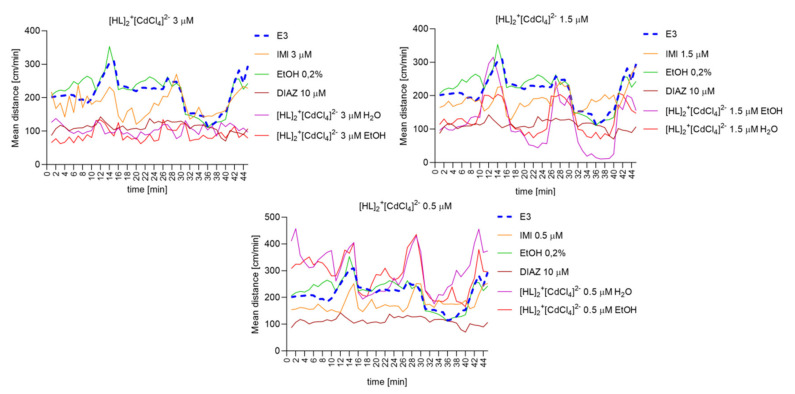
The changes detected during 45 min of the locomotor activity test (three 15- minutes light–dark sessions: 0–10-min of illumination followed by 11–15 min of darkness, 16–25 min of illumination followed by 26–30 min of darkness, and 31–40 min of illumination followed by 41–45 min of darkness) after 40 min of continuous illumination) under the influence of different concentrations of [HL]_2_^+^[CdCl_4_]^2−^ in the solvent-dependent and light–dark-dependent manner of changes.

Repeated measure one-way ANOVA analysis (Figure 14) revealed significant difference in mean distance cm/min as a result of a treatment: 3 µM: (F (2.063, 90.76) = 171.8, *p* < 0.0001), and the time (F (44, 220) = 3.221, *p* < 0.0001); 1.5 µM: (F (2.511, 110.5) = 77.34, *p* < 0.0001) and the time (F (44, 220) = 6.053, *p* < 0.0001; 0.5 µM: (F (2.728, 120.0) = 134.1, *p* < 0.0001), and the time (F (44, 220) = 5.312, *p* < 0.0001).

## 3. Materials and Methods

### 3.1. Chemistry

All chemicals used for the synthesis were purchased from the companies: Sigma-Aldrich, AlfaAesar, POCH and used without further purification. FTIR spectra were recorded with an IRTracer-100 Shimadzu Spectrometer (4000–600 cm^−1^) with an accuracy of recording 1 cm^−1^, using KBr pellets. The thermolysis of compounds in air atmosphere was studied by TG-DTG-DTA techniques in the range of temperature 25 to 800 °C at a heating rate of 10 °C min^−1^; TG, DTG, and DTA curves were recorded on a Netzsch TG 209 apparatus under air atmosphere, v = 20 mL min^−1^, using ceramic crucibles. As a reference material, ceramic crucibles were used.

### 3.2. X-ray Diffraction Analysis

Crystal structures were determined by the Single Crystal X-ray analysis method. The crystals to be measured were selected from the product obtained by described synthesis. X-ray data were collected on the XtaLAB Synergy, Dualflex, Pilatus 300K diffractometer apparatus (Rigaku Corporation, Tokyo, Japan) equipped with the PhotonJet microfocus X-ray tube apparatus (Rigaku Corporation, Tokyo, Japan). Data reduction was performed with CrysAlisPro (Agilent Technologies UK Ltd., Yarnton, England) [25]. The structures were refined in ShelXL [26]. Molecular structures and packing diagrams were drawn using Mercury [27]. Molecular geometry parameters were computed with publCIF [28].

CCDC: Deposition Number 2090557-2090558 contains the supplementary crystallographic data for this paper. The data are provided free of charge by The Cambridge Crystallographic Data Centre via www.ccdc.cam.ac.uk/structures. Deposited: 06/17/2021.

### 3.3. Magnetic Studies

Magnetization measurements were carried out using a superconducting quantum interference device (SQUID) magnetometer with Quantum Design 7T magnetic property measurement system (MPMS XL-7) in the magnetic field range 0.0 T < H < 7.0 T and temperature interval 2.0 K < T < 300.0 K. The magnetic susceptibility χ of the samples was calculated by normalizing the measured magnetic moment by the applied magnetic field.

### 3.4. Biological Assays

Zebrafish Husbandry and Egg Collection: Danio rerio (Zebrafish, wild type zebrafish strain (AB strain)) stocks from the local husbandry in the Experimental Medicine Centre in Lublin, (Medical University of Lublin, Poland) were housed at 28.5 °C, on a 14/10 h light/dark cycle, in the embryo medium (E3): pH 7.1–7.3; 17.4 µM NaCl, 0.21 µM KCl, 0.12 µM MgSO_4_ and 0.18 µM Ca(NO_3_)_2_ under standard aquaculture conditions during the whole time of the experiment. The experiment started with eggs (0 days post-fertilization, dpf) and finished with 5 dpf larvae. Larvae were killed by immersion in 15 μM tricaine solution. All experiments were conducted following the National Institute of Health Guidelines for the Care and Use of Laboratory Animals and the European Community Council Directive for the Care and Use of Laboratory Animals of 22 September 2010 (2010/63/EU).

Tested concentrations: Stock solutions were prepared using two different solvents: ultrapure water and absolute ethanol (Sigma, Burlington, MA, USA, 99.8%). [HL]_2_^+^[CuCl_4_]^2−^ (100 mM in water, 50 mM in ethanol), [HL]_2_^+^[ZnCl_4_]^2−^ (50 mM in water and ethanol), and [HL]_2_^+^[CdCl_4_]^2−^ (12.2392 mM in water and ethanol) stock solutions were dissolved by sonification. Stock solutions were kept at 0–4 °C and were used to prepare all working dilutions of the tested compounds. In the first part of the project, toxic concentrations were established for all tested compounds. Compounds were tested in the following concentrations: 100 μM, 50 μM, 25 μM, 12.5 μM, 6.25 μM, 3.125 μM, 2 μM ([HL]_2_^+^[CuCl_4_]^2−^), 1.25 μM, and 0.125 μM. In the second part of the project, maximum tolerated doses were assessed and three non-toxic concentrations were applied to the behavioral protocol. Diazepam (Relanium, 5 mg/mL, solution for injection, Polfa, Warsaw, Poland) stock solution (100 mM) in E3 and imipramine hydrochloride (Merck, Kenilworth, NJ, USA) stock solution (3 mM) in E3 were prepared for locomotor activity assay. The dose of diazepam (10 µM) for behavioral tests was chosen based on literature data [11].

Fish Embryo Acute Toxicity Test (FET) and Lethal Concentration 50 (LC 50) determination: The FET was performed according to the requirements described by Busquet et al., (2013) in the OECD guidelines for the testing of chemicals 236—Fish Embryo Acute Toxicity (FET) Test with minor modifications. On the first day, newly fertilized zebrafish eggs were exposed to the tested chemicals’ solutions for 3 h. After this time, the mortality was analyzed and only well-developed embryos were selected (20 per group/5 per well in 24-well plaque). For the next 96 h, the malformations appearance, heart rate, hatching, and mortality rate were observed. Every 24 h, all solutions were replaced by freshly prepared dilutions of the tested compounds. Two control groups (20 eggs/group) were immersed in the E3 solution and ethanol in the E3 solution (0.2%). The maximum tolerated concentration of ethanol was established in the preliminary studies (0.2%). The test was considered valid when less than 90% of embryos were found dead until the end of 96 h of exposure. The maximum tolerated doses and LC 50 were calculated.

Locomotor activity and anxiety protocol: 24 5 dpf-larvae were used for each concentration (8 larvae/plague, 3 repetitions). Larvae were bathed in the freshly prepared solutions (3 µM, 1.5 µM, 0.5 µM) of tested compounds and imipramine or diazepam (10 µM) for 30 min before the test in the dark (28 °C). Twenty-four multi-well plates were used for the assays and only one larva was placed in each well in the 1 mL of tested solution. Plates were situated in the zebra box for 95 min; that included the acclimatization part (min 0–10), with the light on, and the visual motor challenge part lasting from 11 min to 95 min. The second part was divided into the continuous illumination phase lasting for 40 min (evaluation of the spontaneous locomotor activity and thigmotaxis behavior, before applying the factor causing the anxiety), and light/dark transition where larvae were subjected to three cycles (the 10-min continuous light and the 5-min dark phase (anxiogenic factor)).

Statistical Analysis: GraphPad Prism (8.0.1) software was used to perform one-way ANOVA analyses with posthoc Tukey’s test to evaluate spontaneous locomotor activity (light–dark phase) and the hatching rate. For two-way ANOVA analysis, two main contributing factors were used: treatment (control groups, and [HL]_2_^+^[CuCl_4_]^2−^, [HL]_2_^+^[ZnCl_4_]^2−^, [HL]_2_^+^[CdCl_4_]^2−^), and solvent differences (water, ethanol 0.2%) or treatment (control groups, and [HL]_2_^+^[CuCl_4_]^2−^/H_2_O, [HL]_2_^+^[CuCl_4_]^2−^/EtOH [HL]_2_^+^[ZnCl_4_]^2−^/H_2_O, [HL]_2_^+^[ZnCl_4_]^2−^/EtOH, [HL]_2_^+^[CdCl_4_]^2−^/H_2_O, [HL]_2_^+^[CdCl_4_]^2−^/EtOH) and the phase (light/dark). Post-hoc Bonferroni’s multiple comparison tests were performed. Nonlinear fit analysis was used to evaluate the LC 50. Repeated measure one-way ANOVA analysis was used to evaluate locomotor activity (0–45 min, light–dark phases). A *p*-value of <0.05 was considered statistically significant. Results are presented in graphs as mean ± SEM.

## 4. Conclusions

In this paper, we have thoroughly investigated the influence of incorporation of different cations into the imipramine system on its physicochemical and biological properties. We have synthesized three new compounds, which have been structurally and thermally described. All three compounds are isostructural with approximately the same lattice parameters. FTIR spectra are in good agreement with structures measured using the SC-XRD method. All compounds are stable at room temperature and decompose gradually along with the increasing temperature. Results suggest that [HL]_2_^+^[CuCl_4_]^2−^ compound has a magnetic response consistent with one unpaired magnetic center of spin 1/2 per molecule. The signal can also originate from Cu(II) on the Cu_2_Cl_4_ structures in the material. Meanwhile, [HL]_2_^+^[ZnCl_4_]^2−^ and [HL]_2_^+^[CdCl_4_]^2−^ appear to be non-magnetic. Measurements allowed an estimation of paramagnetic impurities in the latter below 0.2% and 10 ppm on the [HL]_2_^+^[ZnCl_4_]^2−^ and [HL]_2_^+^[CdCl_4_]^2−^ samples, respectively.

The FET did not show any malformations in the process of Zebrafish development after application of the complexes for 96 h. However, a higher mortality rate was observed after application of [HL]_2_^+^[CuCl_4_]^2−^, [HL]_2_^+^[ZnCl_4_]^2−^, and [HL]_2_^+^[CdCl_4_]^2−^ in water when compared to the mortality rate caused by compounds in ethanol. The lowest mortality rate after 96 hpf was obtained for [HL]_2_^+^[CdCl_4_]^2−^ and the highest for [HL]_2_^+^[CuCl_4_]^2−^ in water. The safest dose for [HL]_2_^+^[CuCl_4_]^2−^ was 3 µM, which is low and makes constraints for further investigations of this compound. [HL]_2_^+^[ZnCl_4_]^2−^ and [HL]_2_^+^[CdCl_4_]^2−^ occurred to be less toxic giving a low percentage of deaths for doses equal to and lower than 6.25 µM and 25 µM, respectively. Nevertheless, the doses equal to and higher than 6.25 µM of [HL]_2_^+^[ZnCl_4_]^2−^ in ethanol and 12.5 µM in water, as well as the doses equal to and higher than 12.5 µM [HL]_2_^+^[CdCl_4_]^2−^ in water and 25 µM [HL]_2_^+^[CdCl_4_]^2−^ in ethanol decreased a hatching rate after 96 hpf. Moreover, 25 and 12.5 µM of [HL]_2_^+^[CdCl_4_]^2−^ and [HL]_2_^+^[ZnCl_4_]^2−^ in both solvents occurred to decrease the heart rate after 96 hpf, while the lower doses did not affect the activity of the Zebrafish heart. After 48 hpf, [HL]_2_^+^[ZnCl_4_]^2−^ caused dose and solvent-dependent changes in the heart rate which were stabilized after 96 hpf. Moreover, [HL]_2_^+^[CdCl_4_]^2−^ applied at the doses of 6.25 µM in water and 3.125 µM in ethanol caused disruptions in the heart rate compared with the control groups which were stabilized after 96 hpf. This may suggest the influence of these compounds on the heart activity and developing abilities in the early stages of the development, but it requires confirmation in further studies.

[HL]_2_^+^[CuCl_4_]^2−^ affected the hatching rate stronger than the other complexes and inhibited it in a dose equal to and higher than 0.125 µM in ethanol and 1.25 µM in water after 96 hpf. Moreover, there were abnormalities in the heart activity associated with the decrease of the heart rate only after 96 hpf when [HL]_2_^+^[CuCl_4_]^2−^ was applied, suggesting the developing cardiotoxicity of the complex. The solvent-dependent differences in the heart rate were also visible.

Water solutions caused deaths of embryos in the early stage of development when compared with ethanol solutions. We may suggest that ethanol formulates safer compound–solvent complexes than water. Interestingly, the deaths of dividing cells appeared also after 3 h after [HL]_2_^+^[CuCl_4_]^2−^ application in the doses 100–6.25 µM in water and 25–12.5 µM in ethanol and 3 h after application of 100 µM of [HL]_2_^+^[ZnCl_4_]^2−^ in water, which may suggest the embryotoxic effect of these compounds in specified concentrations. This confirms the toxic effect of [HL]_2_^+^[CuCl_4_]^2−^ compounds and suggests the influence of mentioned above doses on the developing cell processes. We may claim that higher doses of the tested compounds could have a teratogenic effect on developing organisms.

All tested compounds presented a dose-dependent decrease in the spontaneous locomotor activity where the highest dose 3 µM decreased and the lowest 0.5 µM increased the locomotor activity when compared with control groups after 30-min bathing. The manner of changes was similar for each compound and was not solvent-dependent.

After the application of 3, 1.5, and 0.5 µM of [HL]_2_^+^[CuCl_4_]^2−^ in ethanol, locomotor activity in the light phase stayed on the same level which was lower than the control groups. Moreover, the overall locomotor activity in the dark phase after dissolving [HL]_2_^+^[CuCl_4_]^2−^ in water was lower than in ethanol and lower than in the control group. Studies showed that the acute application of small doses of ethanol influences the level of anxiety-like behavior in Zebrafish [29,30]. In our study, imipramine–metallic ion compounds were also dissolved in ethanol. For this reason, the activity of these solutions could result in either higher dissolving abilities or applied solvent. This may suggest that ethanol as a solvent for this compound enhances the anxiogenic activity of the [HL]_2_^+^[CuCl_4_]^2−^ in a dose-dependent manner, while water intensifies the anxiolytic activity of the tested compounds. The increase in locomotor activity in the dark phase of the experiment by [HL]_2_^+^[CuCl_4_]^2−^ compound is not only dose-dependent, but also solvent-dependent. 

Taken together, we can suggest that the highest doses (3 µM) may have anxiolytic activity associated with the higher locomotor activity when compared with the control group in the dark phase versus the lowest doses (0.5 µM) of anxiogenic activity. The activity of 3 µM of [HL]_2_^+^[ZnCl_4_]^2−^ and [HL]_2_^+^[CdCl_4_]^2−^ complexes in water and ethanol were similar to the effect obtained after 10 µM of diazepam (the anxiolytic agent). That may confirm our conclusions about the anxiolytic activity of this dose without solvent dependence. Each compound caused a dose-dependent manner of changes in the level of locomotor activity presented in the dark phase without a solvent dependence. 

The activity of each dose of complexes differed from the effect caused by the imipramine which suggests that the addition of the cations of Cu^2+^, Zn^2+^, or Cd^2+^ modified imipramine’s influence on the CNS and anxiety-like behavior. The highest safe doses of the tested compounds and [HL]_2_^+^[CuCl_4_]^2−^ in water showed similar effects to diazepam (10 µM) what may suggest their anxiolytic effect. However, the underlying mechanism of this activity associated with a dose- and/or solvent-dependent manner of changes remains unknown. Thus, further biochemical studies are required to investigate the level of neurotransmitters after complex application.

## Figures and Tables

**Table 1:** Crystal data for [HL]_2_^+^[CuCl_4_]^2−^ and [HL]_2_^+^[ZnCl_4_]^2−^ compounds.

	[HL]_2_^+^[CuCl_4_]^2−^	[HL]_2_^+^[ZnCl_4_]^2−^
Crystal data		
Chemical formula	Cl_4_Cu·2(C_19_H_25_N_2_)	Cl_4_Zn·2(C_19_H_25_N_2_)
*M*_r_	768.16	769.99
Crystal system, space group	Orthorhombic, *Pbca*	Orthorhombic, *Pbca*
Temperature (K)	100	294
*a*, *b*, *c* (Å)	18.8810 (3), 11.6982 (2), 33.9528 (5)	19.2344 (5), 11.6465 (3), 34.6682 (8)
*V* (Å^3^)	7499.3 (2)	7766.1 (3)
*Z*	8	8
Radiation type	Mo *K*α	Mo *K*α
No. of measured, independent and observed [*I* > 2σ(*I*)] reflections	37,197, 37,197, 29,899	124,476, 12,164, 7813
*R*(F^2^ > 2σ(*F*^2^)), *wR*(*F*^2^), *S*	0.048, 0.121, 1.04	0.044, 0.108, 1.01
No. of reflections	37,197	12,164
No. of parameters	465	500
Δ〉_max_, Δ〉_min_ (e Å^−3^)	0.77, −0.72	0.25, −0.34

## Data Availability

Not applicable.

## Supplementary Materials

The following are available online at https://www.mdpi.com/article/10.3390/ijms222312909/s1.
