# Supplementary figures and images for: Influence of Incorporation of Different dn-Electron Metal Cations into Biologically Active System on Its Biological and Physicochemical Properties
Source: Int J Mol Sci. 2021 Nov 29;22(23):12909. doi: 10.3390/ijms222312909 (PMC8657940; doi:10.3390/ijms222312909)

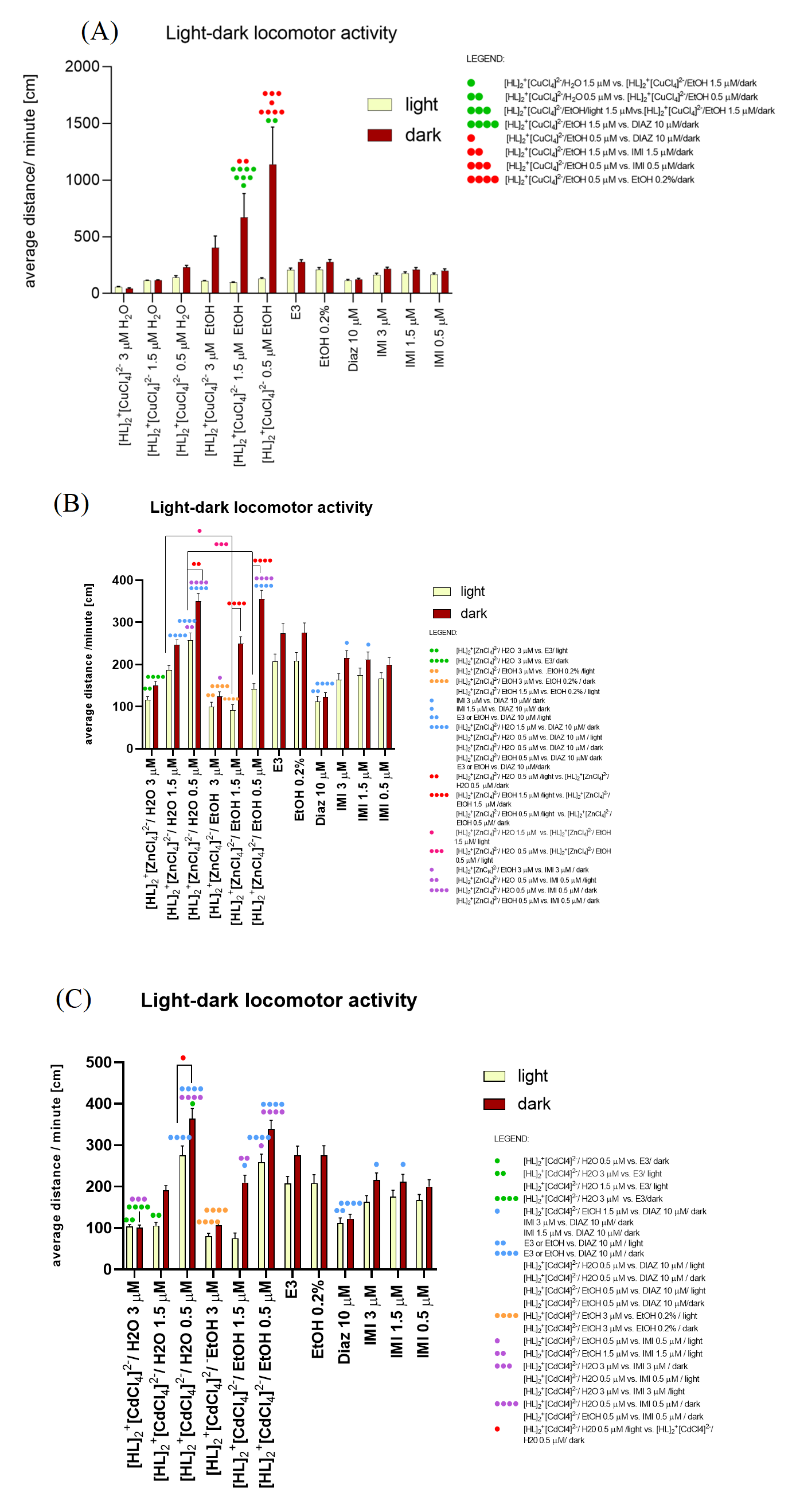

Supplement: Supplementary file 1 [file ijms-22-12909-s001.zip › Supplementary Material/Figure S10.png]

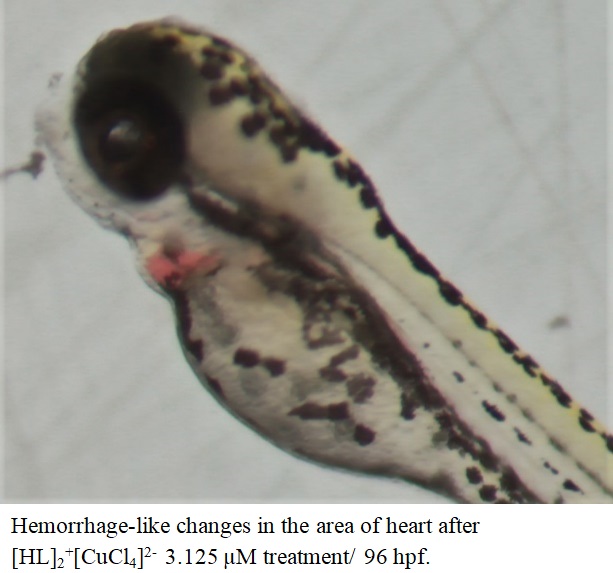

Supplement: Supplementary file 1 [file ijms-22-12909-s001.zip › Supplementary Material/Figure S1.jpg]

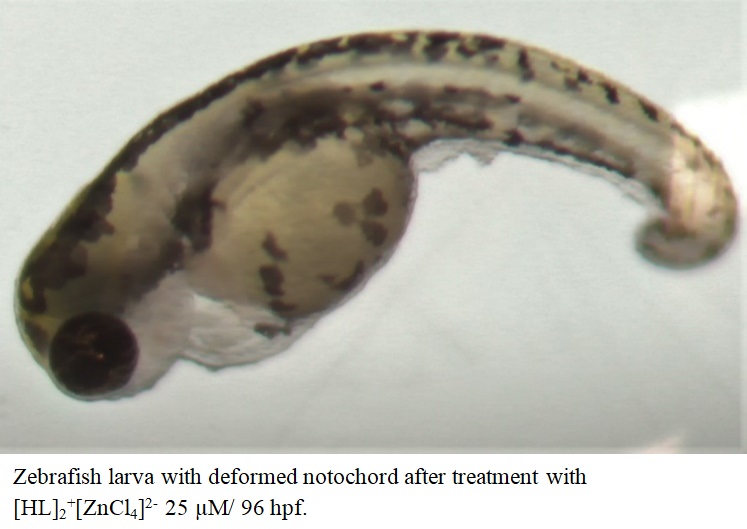

Supplement: Supplementary file 1 [file ijms-22-12909-s001.zip › Supplementary Material/Figure S2.jpg]

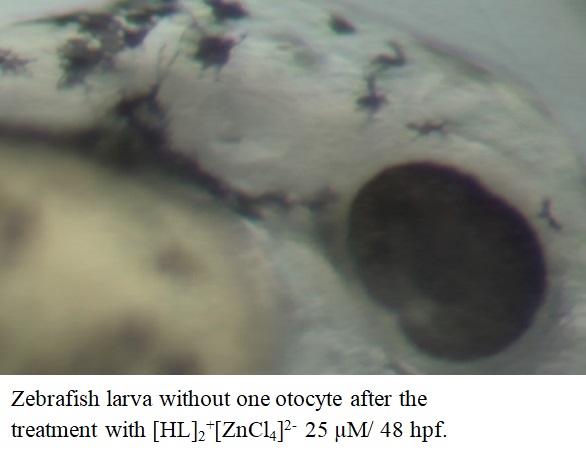

Supplement: Supplementary file 1 [file ijms-22-12909-s001.zip › Supplementary Material/Figure S3.jpg]

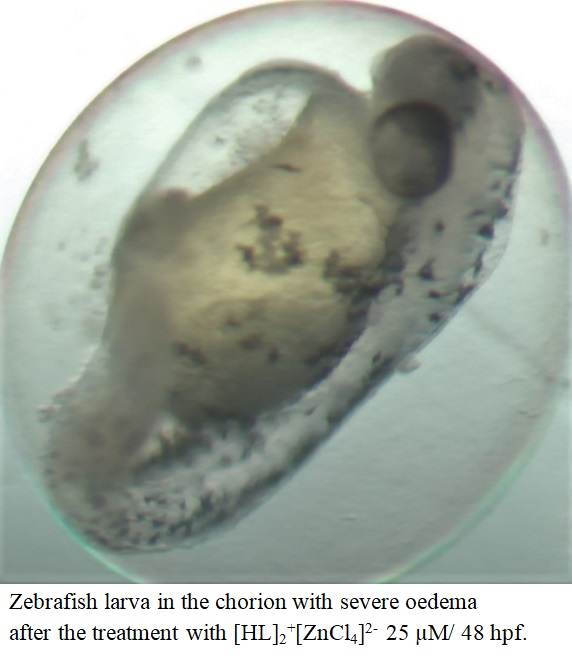

Supplement: Supplementary file 1 [file ijms-22-12909-s001.zip › Supplementary Material/Figure S4.jpg]

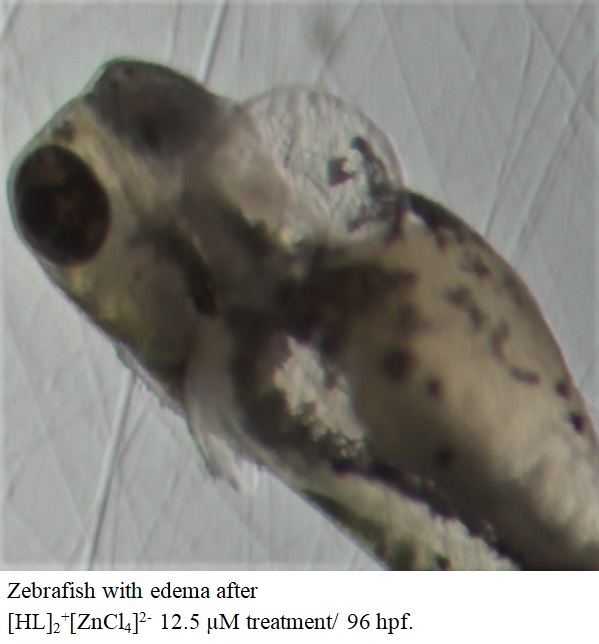

Supplement: Supplementary file 1 [file ijms-22-12909-s001.zip › Supplementary Material/Figure S5.jpg]

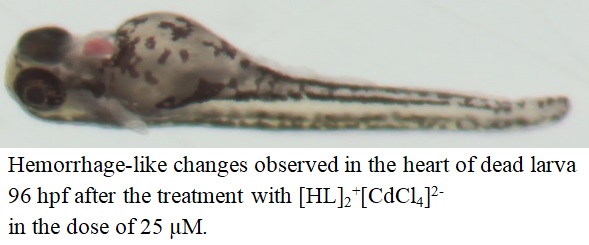

Supplement: Supplementary file 1 [file ijms-22-12909-s001.zip › Supplementary Material/Figure S6.jpg]

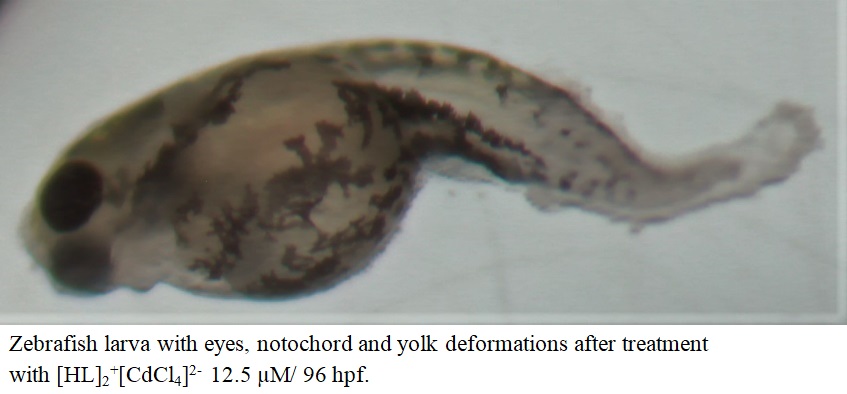

Supplement: Supplementary file 1 [file ijms-22-12909-s001.zip › Supplementary Material/Figure S7.jpg]

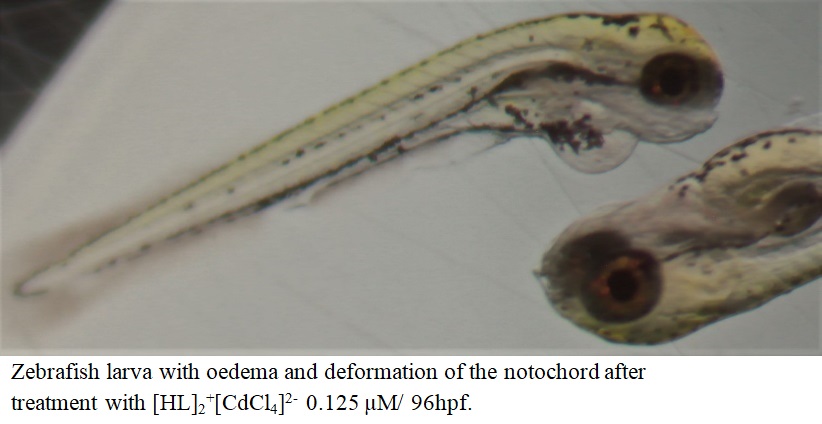

Supplement: Supplementary file 1 [file ijms-22-12909-s001.zip › Supplementary Material/Figure S8.jpg]

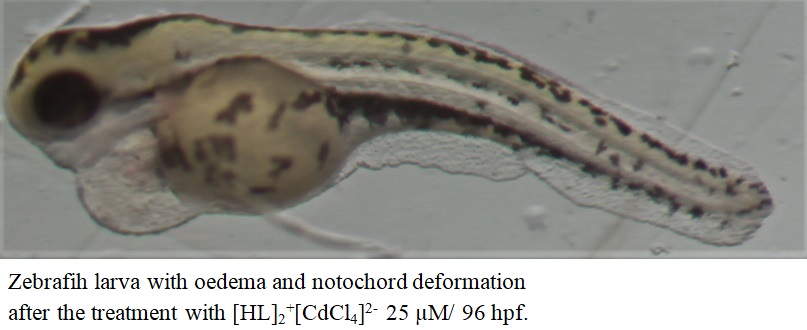

Supplement: Supplementary file 1 [file ijms-22-12909-s001.zip › Supplementary Material/Figure S9.jpg]
